# Ancient DNA from South-East Europe Reveals Different Events during Early and Middle Neolithic Influencing the European Genetic Heritage

**DOI:** 10.1371/journal.pone.0128810

**Published:** 2015-06-08

**Authors:** Montserrat Hervella, Mihai Rotea, Neskuts Izagirre, Mihai Constantinescu, Santos Alonso, Mihai Ioana, Cătălin Lazăr, Florin Ridiche, Andrei Dorian Soficaru, Mihai G. Netea, Concepcion de-la-Rua

**Affiliations:** 1 Department of Genetics, Physical Anthropology and Animal Physiology, University of the Basque Country UPV/EHU, Bizkaia, Spain; 2 National History Museum of Transylvania, Cluj-Napoca, Romania; 3 “Francisc I. Rainer" Institute of Anthropology, Romanian Academy, Bucharest, Romania; 4 Department of Medicine, Radboud University Nijmegen Medical Centre, Nijmegen, The Netherlands; 5 National History Museum of Romania, Bucharest, Romania; 6 Oltenia Museum Craiova, Craiova, Romania; 7 Radboud Center for Infectious Diseases, Radboud University Nijmegen Medical Centre, Nijmegen, The Netherlands; IPATIMUP (Institute of Molecular Pathology and Immunology of the University of Porto), PORTUGAL

## Abstract

The importance of the process of Neolithization for the genetic make-up of European populations has been hotly debated, with shifting hypotheses from a demic diffusion (DD) to a cultural diffusion (CD) model. In this regard, ancient DNA data from the Balkan Peninsula, which is an important source of information to assess the process of Neolithization in Europe, is however missing. In the present study we show genetic information on ancient populations of the South-East of Europe. We assessed mtDNA from ten sites from the current territory of Romania, spanning a time-period from the Early Neolithic to the Late Bronze Age. mtDNA data from Early Neolithic farmers of the Starčevo Criş culture in Romania (Cârcea, Gura Baciului and Negrileşti sites), confirm their genetic relationship with those of the LBK culture (*Linienbandkeramik Kultur*) in Central Europe, and they show little genetic continuity with modern European populations. On the other hand, populations of the Middle-Late Neolithic (Boian, Zau and Gumelniţa cultures), supposedly a second wave of Neolithic migration from Anatolia, had a much stronger effect on the genetic heritage of the European populations. In contrast, we find a smaller contribution of Late Bronze Age migrations to the genetic composition of Europeans. Based on these findings, we propose that permeation of mtDNA lineages from a second wave of Middle-Late Neolithic migration from North-West Anatolia into the Balkan Peninsula and Central Europe represent an important contribution to the genetic shift between Early and Late Neolithic populations in Europe, and consequently to the genetic make-up of modern European populations.

## Introduction

The fundamental question of the relative contribution of Palaeolithic hunter-gatherers and Neolithic farmers regarding the genetic heritage of present-day Europeans has been hotly debated. Three events are believed to have had a major impact in the present-day genetic variability of Europeans: the expansion of modern humans from Africa through the Middle-East some 46.000 years ago, the repopulation of Europe after the Last Glacial Maximum between 27.000 and 16.000 years ago, and the arrival of the Neolithic culture from Anatolia between 9.000 and 5.000 years ago [1].

The studies by Menozzi, Piazza and Cavalli-Sforza on classical genetic markers, more than three decades ago, described a South-East to North-West PC1 component that was interpreted as a demic diffusion of Neolithic farmers from the Middle East into Europe [2–3]. These data were however challenged by DNA analysis from present-day populations ([4–7] among others) and more recently by ancient DNA (aDNA) studies based on mitochondrial DNA (mtDNA) [8–23]. aDNA studies of hunter-gatherers revealed a high genetic homogeneity in the pre-Neolithic groups throughout Europe, whether from Scandinavia [8–10], Central Europe [11] or the Iberian Peninsula [12–13]. The analysis of aDNA from Early European farmer groups of the Linear Pottery Culture (LPC, also known as *Linienbandkeramik Kultur* or LBK) in Central Europe suggested a genetic discontinuity in Central Europe and favored instead of a process of Neolithic transition through a demic diffusion model (DD) [14–15]: this view was based on a high frequency of the N1a haplogroup (about 15%) in the LBK farmers [15], absent in hunter-gatherers in this same region [11] and almost nonexistent (0.2%) in the present-day European populations [15]. On the other hand, these first farmers shared an affinity with the modern-day populations from the Near East and Anatolia, supporting a major genetic input from this area during the advent of farming in Europe [15]. Studies of other Neolithic sites in the North of France, Hungary and the Northeast of Iberian Peninsula also supported this view [16–18]. However, an ancient mtDNA study of a Neolithic site in the Mediterranean region of Europe, namely in the Iberian Peninsula, led to the proposal of a dual model for explaining the Neolithic dispersion process in Europe: DD in Mediterranean area and CD in Central Europe [19].

On the other hand, it has also been proposed that the mtDNA variability in the Cantabrian Fringe (nine archaeological sites of both Hunter-Gatherers and Farmers) is best explained by a model of random rather than clinal dispersal of Neolithic farmers in Europe, with different genetic influence in different geographical regions and in different periods of time [12]. In regard to Central Europe, a comprehensive study on mtDNA from archaeological sites spanning from the Early Neolithic to the Early Bronze Age identified four marked genetic shifts during the Neolithic period. This diachronic study reported a marked genetic shift between the Early/Middle and Late Neolithic populations, with a key role for Late Neolithic cultures in shaping the genetic diversity of modern central Europe genetic diversity [21]. How did this marked genetic shift between Early/Middle and Late Neolithic could occur in a relatively limited period of time is unclear.

Additionally, a recent mtDNA study on a sample of 15 Near Eastern farmers has revealed genetic affinities between these earlier farmer communities and modern populations from Cyprus and Crete, suggesting that the Neolithic was first introduced into Europe through pioneer seafaring colonization [22].

Finally, the study of the genomes of a 7,000-year-old farmer from Germany and eight ~8,000-year-old hunter-gatherers from Luxembourg and Sweden have shown that most present-day Europeans derive from at least three highly differentiated populations. Besides, authors have proposed that early European farmers had a ~44% ancestry from a ‘basal Eurasian’ population [23].

While much has been learned by the aforementioned studies, two crucial aspects have not been taken into consideration. Firstly, archaeological data show that the Neolithic expansion from Anatolia was not a single event but was represented by several waves of migrants [24]. In this respect the Proto-Sesklo culture in Greece, from which directly Starčevo-Criş in the North Balkans and indirectly LBK in Central Europe originate [25–26] represents only the first great wave of Neolithisation of Europe [27]. A later great wave of migration from North-West Anatolia led to important cultures of South-Eastern Europe such as Vinča and Boian cultures [28]. Secondly, there is a total absence of aDNA data from South-East Europe in the current models.

**Fig 1:**
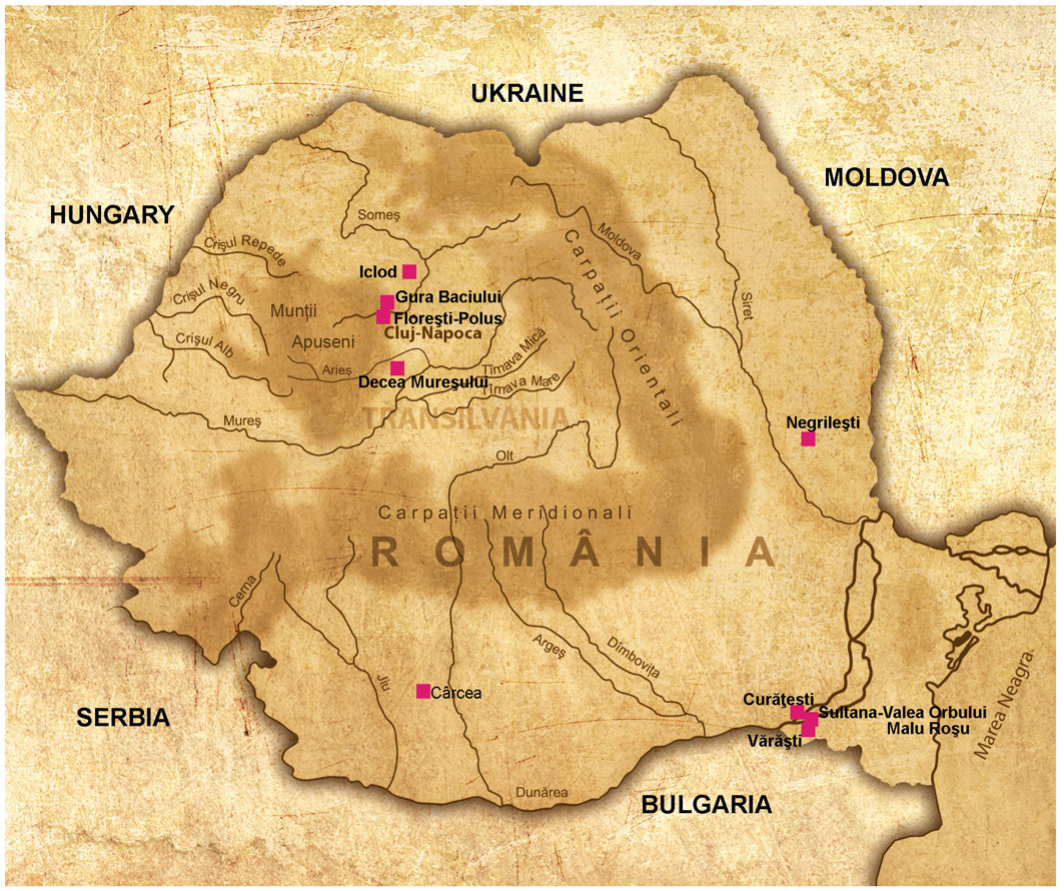
Geographic location of ten Romanian sites analyzed in the present study. (The figure has been provided by M. Rotea and T. Károly).

In the present study we have assessed the mtDNA variability from 63 individuals recovered from 10 archaeological sites in Romania spanning a period of five and a half millennia (c. 6300–1100 cal BC) between the Early Neolithic to the Late Bronze Age in Romania (Table 1, Fig 1). This is a strategic area of South-East Europe, from which different prehistoric human groups have passed and later spread throughout Europe. These sites encompass several major cultural events: i. the first Neolithic complex of the Gura Baciului- Cârcea group (also called Precriş culture) of Starčevo-Criș culture, which has the same origin in the Proto-Sesklo culture and it is partially contemporary with LBK culture in Central Europe; ii. the Boian, Zau and Gumelniţa cultures, that represent a continuum of a second migration in the Middle/Late Neolithic and Eneolithic, which has its origin in North-West Anatolia (Demircihoyuk) through East Bulgaria [28–29]; iii. the Eneolithic complex of Decea Mureşului, that represents a possible eastern migration [30–32]; and iv. the Early and Late Bronze Age complex of Floreşti-Polus, that represents new migratory movements most likely originating in the North steppes of the Black Sea [29]. The aim of the study is to shed light on the genetics of the different waves of migration of Neolithic and Bronze Age populations penetrating Europe from Anatolia and the steppes north of the Black Sea. We also assess the genetic impact of prehistoric events in the genetic composition of the present-day European populations.

**Table 1:** Prehistoric samples from Romania analysed in the present study: Chronology, Cultural stages (also in Supporting Information S1 Table), Archaeological sites and Sample size (I.D.: Identification name; N analysed: Number of individuals analysed; N rep: number of individuals with reproducibility results).

Chronology and culture	Site	I.D.	N analysed	N rep
**Early Neolithic (E_NEO) (6500–5500 BC) (Cârcea/Gura Baciului/Precriş Culture)**	Gura Baciului	GB	2	2
	Negrileşti	NE	1	1
	Cârcea	CA	2	2
**Middle/Late Neolithic and Eneolithic (M_NEO) (5500–4500 BC) (Boian-Zau and Gumelniţa cultures)**	Iclod	I	3	3
	Vărăşti	Va/BV	14	14
	Curăteşti	Cu	2	2
	Sultana-Valea Orbului	Su	16	12
	Sultana-Malu Roşu	SMR	10	10
**Eneolithic (Eneol) (4500–3800 BC) (Decea Mureşului culture)**	Decea Mureşului	DM	2	2
**Early Bronze Age (E_BA) (2600–2100 BC) (Copăceni culture)**	Floreşti-Polus	P	2	2
**Late Bronze Age (L_BA) (1500–1100 BC) (Noua culture)**	Floreşti-Polus	P	9	9

## Results

### The mtDNA variability of prehistoric groups from Romania

Ancient DNA analysis was performed from 80 teeth remains belonging to 63 individuals recovered from ten prehistoric sites (Table 1, Fig 1 and S1 Table). We have performed ^14^C dating for eleven human remains from six Romanian sites (S8 Fig). One of the samples was discarded because it provided inconsistent dating, while the others were consistent with the archeological dating. Fifty nine informative mtDNA sequences were obtained from a total of 63 individuals, accounting for an overall efficiency of 93% (4 individuals were discarded due to inconsistent results) (S2 Table, Supporting Information). A number of individuals (17%) has been replicated independently, which consisted in performing the extraction, amplification and sequencing of two samples from the same individual by different researchers at different periods of time. The number of molecular targets was quantified for each extract by means of RT-qPCR. The results showed that the number of molecules/μl in the extracts ranged between 200–66000 (S2 Table), values falling within the limits proposed for reliable aDNA studies [33].

In order to identify any possible contamination that might have occurred in the different stages of the laboratory work, at least two extraction controls and several PCR negative controls were included in each amplification reaction. The rate of contamination for this analysis was 1.3%.

In addition, a total of 192 PCR products from 26 individuals were cloned, of which a minimum of ten clones per PCR product were selected and sequenced (S6 Table). The results were used to determine the degree of coincidence between the consensus sequence of the clones and the sequence obtained by direct sequencing. A mean of 8.20 mutations per fragment cloned (~100 pb) were rejected as these mutations were found uniquely in different clones. These mutations have been considered as artefacts resulting from post-mortem damage to aDNA.

### Early Neolithic: Starčevo Criş culture

The mtDNA variability observed in the samples from Early Neolithic in Romania (E_NEO) (n = 5, Gura Baciului, Negrileşti, and Cârcea sites), showed five haplotypes (haplotype diversity = 0.99±0.0395) that were assorted into four European haplogroups (H, HV, J and T1a) (Table 2). The haplogroup H is the most frequent in the present-day European populations and the haplogroups J and T1 are suggested to be as markers of the Neolithic diffusion from Near East [5].

**Table 2:** Haplotype (ht) and haplogroup (hg) mtDNA distribution resulting of the analysis of 62 ancient individuals from Romania.

Chronology	Sample	ht	%	hg	%
**Early Neolithic** (E_NEO)	GB2	ht1	20	J	20
	GB3	ht2	20	HV	20
	NE-1	ht 42	20	H	40
	Ca1	ht16	20	H	
	Ca2	ht17	20	T1a	20
**Middle/Late Neolithic and Eneolithic** (M_NEO)	BV1; Va4; Va8; Su7; Su12; Su16; Su9; SMR-1; SMR-3; SMR-6; SMR-8	ht16	27	H	58.5
	BV2	ht18	2.4	H	
	Va3	ht21	2.4	H	
	Va6	ht23	2.4	H	
	Va11	ht27	2.4	H	
	Va12	ht28	2.4	H	
	Su11; SMR-5	ht33	4.8	H	
	Su14	ht35	2.4	H	
	Su15	ht36	2.4	H	
	SMR-4	ht38	2.4	H	
	SMR-7	ht39	2.4	H	
	SMR-9	ht40	2.4	H	
	SMR-10	ht41	2.4	H2	
	Cu1	ht12	2.4	U5	12.2
	Su3	ht13	2.4	U5	
	Su13	ht34	2.4	U	
	Su1	ht30	2.4	U4	
	Su8	ht32	2.4	U5b	
	I8; I9	ht4	4.8	J	12.2
	Va2	ht20	2.4	J	
	Va5	ht22	2.4	J	
	Va9	ht25	2.4	J	
	Cu2	ht29	2.4	K	4.8
	Su4	ht31	2.4	K	
	Va1	ht19	2.4	T1	4.8
	I6	ht3	2.4	T1a	
	Va7	ht24	2.4	W	2.4
	Va10	ht26	2.4	HV0	2.4
	SMR-2	ht37	2.4	R	2.4
**Eneolithic** (Eneol)	DM3	ht5	50	K	100
	DM4	ht6	50	K	
**Early Bronze Age** (E_BA)	P11	ht7	50	K	100
	P12A	ht7	50	K	
**Late Bronze Age** (L_BA)	P24	ht9	12.5	H1	37.5
	P25	ht10	12.5	H	
	P30	ht15	12.5	H	
	P26	ht11	12.5	HV	25
	P29	ht14	12.5	HV	
	P27	ht12	12.5	U5	25
	P28	ht13	12.5	U5	
	P22; P23*	ht8	12.5	W	12.5

(* only considered one sample).

### Middle/Late Neolithic and Eneolithic: Boian, Zau and Gumelniţa cultures

The sample of this chronological sequence from Romania is represented by 41 individuals from five different sites. Boian culture (c. 5300–4500 cal BC) can be framed in the Middle Neolithic period, while Gumelniţa (c. 4500–4000 cal BC) corresponds to the final stage of the Neolithic in Romania, called Eneolithic (also known as Chalcolithic or Copper Age) [28]. Gumelniţa and Boian are two related cultures, having the same area, same type of settlements, economy and burials, being only different in their chronology. Most archeologists believe that these two cultures represent a continuum [28, 34]. The samples from the Iclod site belong to the Zau culture (who is contemporary with both Boian and Gumelniţa). Therefore, we decided to analyse the samples belonging to Boian, Gumelniţa and Zau cultures together: for the sake of simplicity we will call them M_NEO during the population genetic analysis. In addition, no statitically significance differences were found between these sites, supporting the decision to analyse them together. The analysis of their mtDNA variability showed 29 mitochondrial haplotypes (haplotype diversity = 0.8095±0.0052), which were assorted into eight different haplogroups (H, HV, R, J, K, T, U, W) (Table 2). The most frequent is haplogroup H (58.5%), which showed a high diversity including 13 different haplotypes, while the next most frequent haplogroups were U (12.2%) and J (12.2%). Within haplogroup U five different haplotypes can be seen, with four of them corresponding to the subhaplogroups that were frequent in the European hunter-gatherers (U5 and U4). The haplogroups J and T (T1), which have been proposed as genetic markers of the Neolithic demic diffusion from the Near East [5], showed a frequency of 12.2% and 4.8% respectively. These values are similar to those found in modern European populations, and the same was true for the rest of the haplogroups (K, W, HV, R).

### Eneolithic: Decea Mureşului non-indigenous culture

The samples of Eneolithic in Romania were obtained from two individuals recovered from the Decea Mureşului site (samples identified as Eneol) and belonging to a cultural phenomenon known under the same name. Generally, archaeologists consider that the Decea Mureşului culture is the result of a migration of non-indigenous populations coming from the North Pontic steppes [31]. The material culture of these intrusive communities differs fundamentally from that of the local Eneolithic cultures (e.g. Boian, Gumelniţa, Petresti, Cucuteni, Tiazapolgar, etc.) [28]. This is the reason why the samples of the Decea Mureşului culture were analysed separately of other cultures from the same chronological sequence (e.g. the local Gumelniţa culture).

The two different mitochondrial haplotypes obtained in two individuals recovered from the Decea Mureşului cemetery correspond to haplogroup K. These haplotypes are unique, not found in any prehistoric sample, either Romanian or European (Table 2). These mitochondrial DNA haplotypes have only been found, albeit with a low frequency, in the present-day Middle East populations (1%).

### Early and Late Bronze Age: Copăceni and Noua cultures

The two individuals from the Copăceni group, an Early Bronze Age site (E_BA), showed two different haplotypes, which are included in haplogroup K. These haplotypes are common in present-day and ancient European populations. On the other hand, the mtDNA data obtained from Noua Culture, a Late Bronze Age site (L_BA) in Romania, correspond to eight different haplotypes (haplotype diversity = 0.8889±0.0074), assorted into four European haplogroups (H, HV, U5 and W) (haplogroup diversity = 0.8214±0.1007). It should be highlighted that the haplotypes ht12 and ht13 in the L_BA site, belonging to subhaplogroup U5 (one of the most ancient in Europe) were also found in the Middle-Late Neolithic (M_NEO) groups from Romania (Table 2). One of the haplotypes (ht8 corresponding to haplogroup W) was found in two different individuals in the L_BA site (P22 and P23) (Table 2). As archaeological and anthropological context suggested a possible kinship relation between these two individuals, the analysis of five autosomic STRs in the samples was performed (AMG, D13S317, D2S1338, D18S51, D16S5399 AmpFlSTR MiniFiler PCR amplication Kit, Life Technologies); this genetic analysis confirmed that they likely were sister and brother (initially called “Romeo and Juliet” as they were thought to be a young couple of lovers [35]) (S2 Table). For this reason, only one of these two individuals has been included in the diversity and statistical analysis.

### Comparison of ancient and present-day populations from Romania

A pairwise Fst test based on the mitochondrial haplotype variability showed significant differences between ancient (present study) and modern Romanian populations [36] (S3 Table). No conclusions can be drawn for Eneol and E_BA populations due to the small sample size of those groups (n = 2). When the analysis was performed on the mitochondrial haplogroup variability, the M_NEO and present-day Romania (ROM) populations did not show statistical differences. Analysis of Median Joining Network within prehistoric Romanian populations (presented in the S1 Fig), showed that the most frequent haplotype was rCRS (the central node in the Network, ht16 in Table 2), that was shared by individuals from the Early Neolithic (E_NEO), Middle/Late Neolithic and Eneolithic (M_NEO) and present-day (ROM) groups. Two other shared haplotypes in this network were the 16270 (ht13, U5 in Table 2) and 16192–16270 (ht12, U5 in Table 2), polymorphisms that appeared in M_NEO and L_BA groups. The rest of the haplotypes are specific to each archeological/cultural group.

As it can be seen in the network (S1 Fig), the higher haplotype diversity corresponded to mtDNA lineages from Middle/Late Neolithic and Eneolithic (M_NEO), where haplogroup H presented a high frequency and diversity values (S1 Fig). Therefore, a network including the haplotypes of both the M_NEO and the present-day Romanian [36] populations was built in order to analyze the mtDNA variability shared by these two populations (S2 Fig). It can be observed that most of the shared polymorphisms belong to haplogroup H.

### Comparison of ancient populations from Romania with other ancient populations from Europe

#### Early Neolithic from Romania

The first Neolithic inhabitants of Europe are described archeologically as belonging to the Aegean Early Neolithic cultures [27], from which the bearers of both the Starčevo-Criş-Körös complex in Serbia, Romania and Hungary [28, 37] and the Linear Pottery culture in Central Europe (LBK) [21] emerged. No statistical significant differences were found between mtDNA frequency distribution of these two cultures which is in line with the archaeological evidence of a common origin in the Sesklo cultural complex. It is noteworthy to observe that the haplogroup N1a found in the individuals of LBK culture and which is considered a hallmark of the Early Neolithic populations in Central Europe was absent in the Starčevo-Criş culture groups; however, a bias due to the low number of Early Neolithic samples from Romania cannot be excluded as a cause for this difference (S3 Fig).

#### Middle/Late Neolithic and Eneolithic from Romania

The population corresponding to the Boian, Zau and Gumelniţa cultures from Romania studied here (n = 41) was compared with populations of Central Europe represented by the Baalberge, Salzmünde and Bernburg cultures [21], because of their chronological proximity. The S4 Fig shows that both groups share similar frequencies for haplogroups J, R, U and W, whereas important differences were found for haplogroups H (58.5% in Romania and 22% in Central Europe), K (4.8% and 17% respectively) and T (4.8% and 14.8% respectively). Haplogroups N and X were absent in the Middle/Late Neolithic and Eneolithic (M_NEO) Romanian population. This led to statistically significant differences between Romanian and Central European Neolithic populations for both mtDNA haplogroups and haplotypes (p = 0.00000±0.0000). Median Joining Network analysis of the mtDNA haplotypes of M_NEO groups from Romania and Central Europe displayed differences in their haplotypes distributions (S5 Fig). The only shared polymorphisms are those corresponding to the rCRS (central node of the Network) and polymorphisms 16069 (haplogroup J) and 16298 (haplogroup HV).

The mitochondrial haplotypes obtained in two individuals recovered from the Decea Mureşului site belonged to haplogroup K (Table 2). Therefore, we have performed a Median Joining Network for this haplogroup (S6 Fig), which includes all haplotypes corresponding to the ancient populations of Romania (present study), Czech Republic (Vedrovice) [38], Near Eastern [22], as well as present-day populations (Romania, Near Eastern and Eastern Europe). The network showed that the only shared polymorphisms between Decea Mureşului samples and the rest are those of the central node and two other polymorphisms shared with ancient and modern Near Eastern populations.

#### Early and Late Bronze Age from Romania

Important population shifts due to migratory events coming especially from the East occurred in the Bronze Age on the present territory of Romania. The Early Bronze Age II phase of Florești-Polus site is represented by a novel culture (Copăceni group) characterized by the presence of tumuli and megaliths, and associated with the Yamnaya culture from the Crimea/Volga basin [29, 35]. From this stage, only two individuals were available, who showed the same haplogroup K. In contrast, the late phase of Florești-Polus site represents a new migration event related to the Noua-Sabatinovka culture [29, 35]. Therefore we compared the mtDNA haplogroup frequency of L_BA individuals from Polus with a Bronze Age group from Ukraine [39] (S7 Fig). These two Bronze Age populations shared haplogroups H, U and W, with the largest differences referred to the frequency of haplogroup W. The Bronze Age Ukraine population presented the highest mtDNA haplogroup diversity, due most likely to its large sample size. Significant statistical differences between these groups have not been detected.

### Multivariate analysis: Ancient populations from Romania in the context of past and present-day populations

**Fig 2:**
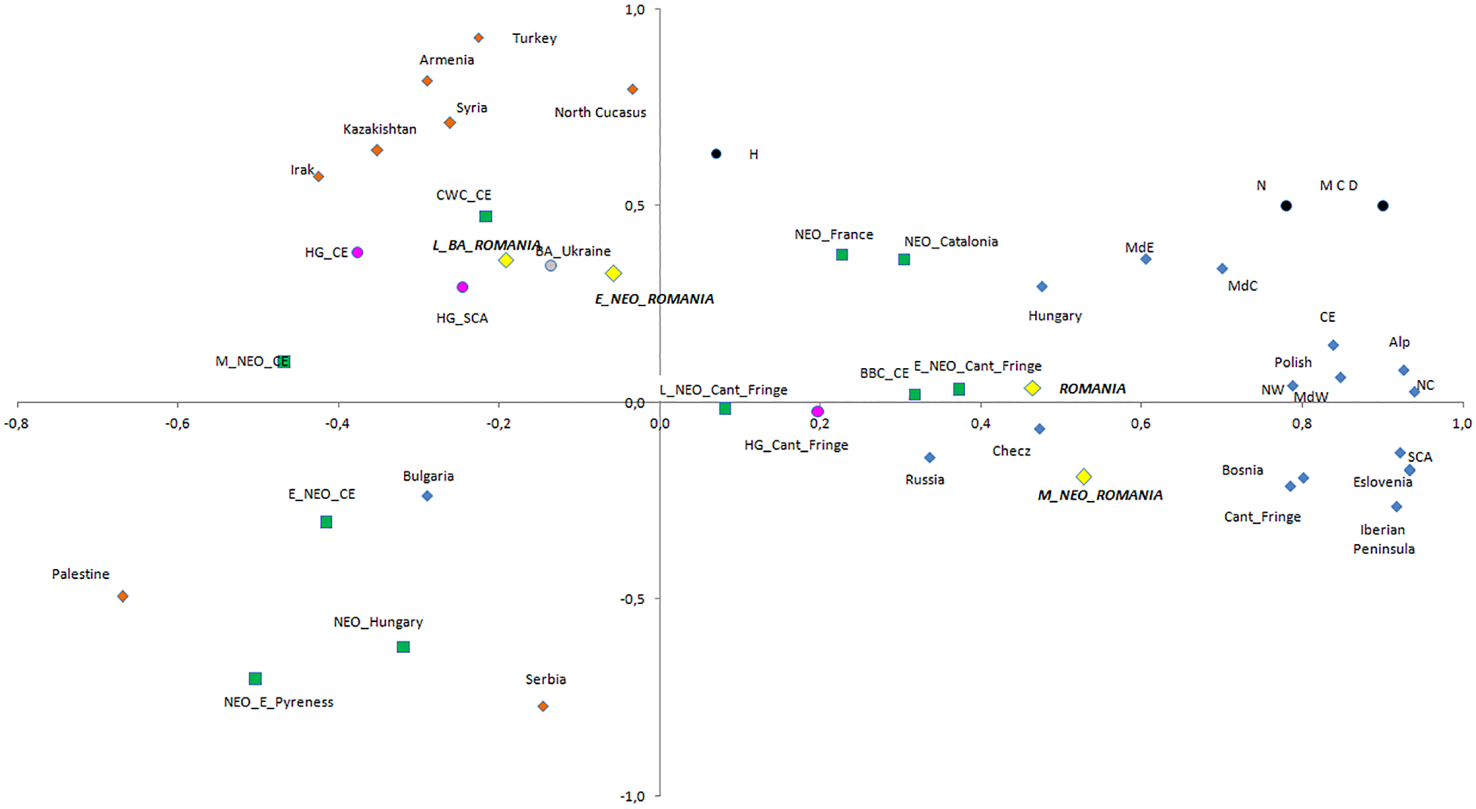
Principal Component Analysis (47% of the total variance) performed considering mtDNA haplogroup frequencies of the ancient and present-day European and Near East populations. In green Neolithic populations, in pink Hunter-Gatherer groups (HG), in yellow ancient and present-day Romania groups, present-day European population in blue and present-day Near East population in orange. Interpretation based on the haplogroup frequency has been written on both PC (Absence of haplogroups D, M, C and N on one side of the first component and absence of haplogroup H on the top of the second component). PC1 represents 30% of variance and PC2 represents 17% of variance.

**Fig 3:**
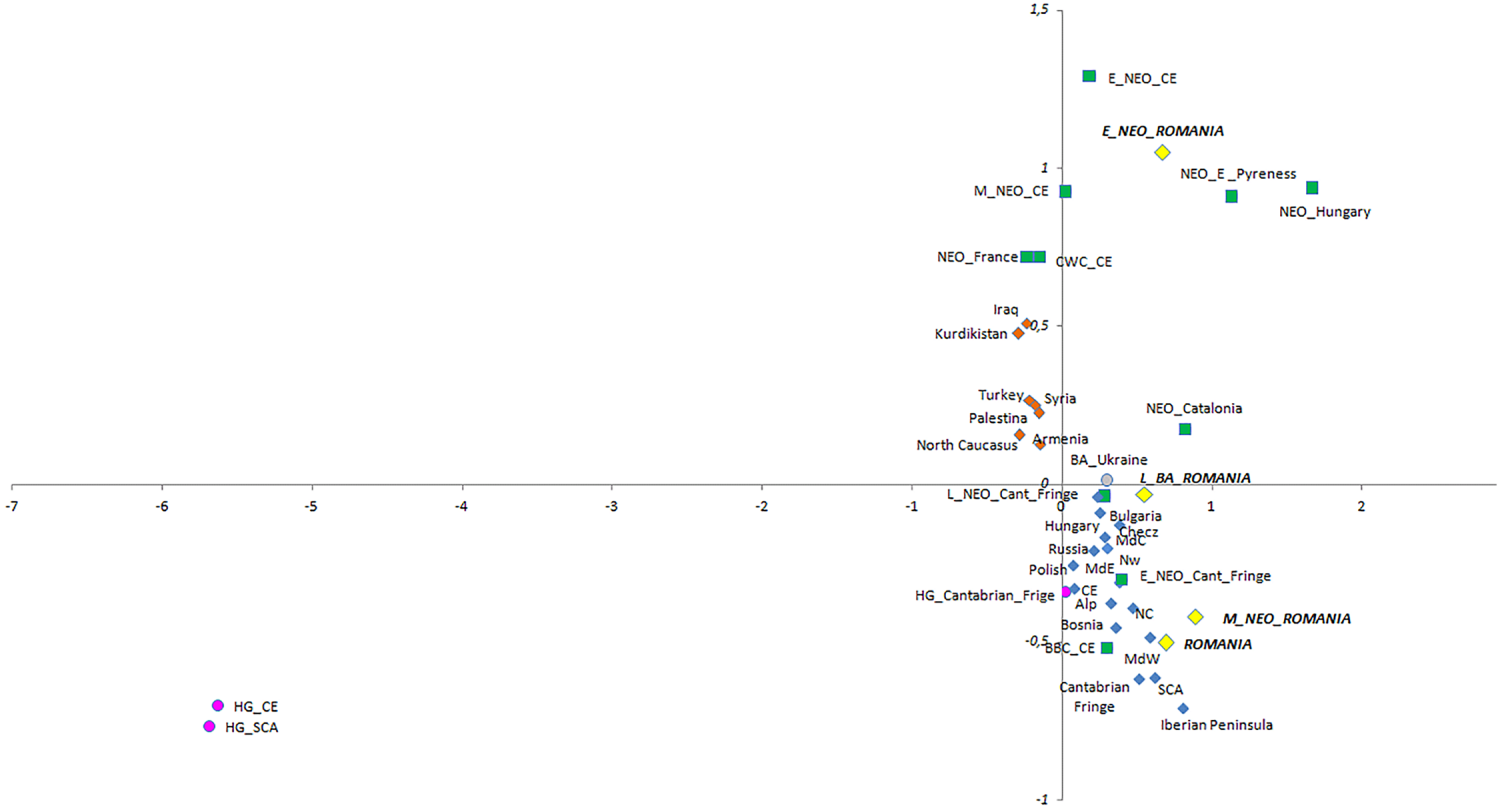
Multidimensional Scaling Analysis performed by haplogroup frequencies of the ancient and present-day European and Near East populations. In green Neolithic populations, in pink hunter-gatherer groups and in yellow ancient and present-day Romanian groups, present-day European population in blue and present-day Near East population in orange. Stress: 0.07553 and RSQ: 0.99071.

We have analyzed the variability of mtDNA haplogroups of ancient Romania groups in the context of other ancient and present-day populations from Europe and Middle East (S4 Table) through two different multivariate analyses: PCA and MDS, Figs 2 and 3. Eneolithic (Eneol) and Early Bronze Age (E_BA) samples from Romania were excluded due to their small sample size. In Figs 2 and 3, the Principal Component Analysis (PCA) and Multidimensional Scaling Analysis (MDS) are shown.

The two first components of the PCA explained 47% of the variance. PC1, representing 30% of the total variance, was related to the haplogroups D, C, M and N (0.962, 0.952, 0.942 and 0.717 respectively). Present-day European populations lay at one end of this axis, the opposite end being associated to the Middle East populations. Prehistoric populations are distributed following a heterogeneous pattern between these two extremes (Fig 2). Early Neolithic (E_NEO) populations from Romania and Central Europe clustered together, while the Middle/Late Neolithic and Eneolithic (M_NEO) population from Romania is not clustered with the Middle Neolithic from Central Europe, but with the modern European populations instead. Overall, a similar conclusion can be inferred from PC2 (17% of the total variance). In this case, the variation is explained by haplogroup H, which had the highest correlation value with this component (0.691). The M_NEO group from Romania showed a high frequency for haplogroup H (58.5%), basically similar to modern Europeans, but different from the Early Neolithic groups from Romania.

Finally, a MDS providing a two-dimensional view of a F_ST_ distances matrix was performed. F_ST_ values were calculated according to the frequency of the mitochondrial haplogroups. The results of this analysis are shown in Fig 3, with a reliable graphic representation of the genetic distances (RSQ of 0.99071 and Stress of 0.07553). As previously shown, hunter-gatherer populations in Scandinavia [8–10] and Central Europe [11, 21] (HG_SCA and HG_CE) are clearly different from all other populations in the analysis. The Early Neolithic groups from Romania and Central Europe [14–15, 21] (E_NEO_Romania and E_NEO_CE) are close despite differences in haplogroup distribution (S3 Fig). In contrast, the Middle Neolithic groups from Romania and Central Europe [21] (M_NEO_Romania and M_NEO_CE) are separated. In the case of Romania, the M_NEO group had a higher genetic distance from the Early Neolithic (E_NEO_Romania) than with the present-day Romanian population. On the contrary, Early and Middle Neolithic populations in Central Europe [21] lay closer to each other than any of them with the present population of the same area. Lastly, the Late Bronze Age Romanian group is closer to Bronze Age from Ukraine than to the M_NEO_Romania (Fig 3).

## Discussion

In the present study we analysed mtDNA from 59 Neolithic, Eneolithic and Bronze Age individuals recovered from ten archaeological sites in Romania (Table 1), in order to evaluate the potential genetic impact of the different ancient populations in South-East Europe spanning from Early Neolithic to the Late Bronze Age (6300 BC to 1100 BC) on the genetic composition of present-day European populations.

### The Early Neolithic farmers in Europe

One of the most hotly debated aspects concerning the origin of Europeans is represented by the relative contribution of Palaeolithic/Mesolithic hunter-gatherers versus the Neolithic farmers for the genetic heritage of modern populations. Two major models for the role of Neolithic farmers and the spread of agriculture have been proposed: a demic diffusion (DD) model and a cultural diffusion (CD) model. In the DD model the Neolithic farmers have a much bigger genetic impact on the make-up of modern Europeans than in de CD model. Although early analyses considered only two models, a number of mtDNA studies in Neolithic populations have indicated a more complex pattern for Neolithic transition. Thus, the random dispersion model proposes that Neolithic farmers had a different impact on the various geographic regions (central Europe, Mediterranean Europe and Cantabrian fringe), at different periods of time [12, 17, 20–23].

Studies from Central and West Europe, especially the analysis of mitochondrial diversity of LBK culture groups, showed no continuity between the first farmers of Europe and the modern Europeans, thus proposing that these Neolithic pioneers had little genetic impact on the current European population [11, 14–15, 21]. This hypothesis is supported by our data, which show a close genetic proximity of Early Neolithic group from Romania (Starčevo-Criş culture) with Early Neolithic populations such as LBK but no genetic continuity with modern Romanian populations (Figs 2 and 3, S3 Table). These data are in line with the idea of a common origin of the LBK and Starčevo-Criş cultures from the Aegean Neolithic cultures of Northern Greece/Thessaly, the first Neolithic complex in Europe [24]. The differential distribution of the mtDNA haplogroup in both Early Neolithic groups (S3 Fig)—highlighting the absence of N1a lineage in E_NEO_Romania, a Neolithic marker in Central Europe—may reflect a differential genetic impact of the Neolithic pioneers in these areas.

### The genetic shift between Early and Middle Neolithic in Europe

A comprehensive study of mtDNA spanning a period from the Early Neolithic to the Bronze Age in Central European populations has been recently completed [21]. In this study, by comparing different Neolithic populations of Central Europe with a Central European metapopulation, the authors proposed four major demographic events. Their analysis supported a model of continuity between Late Neolithic and modern European populations, while Early and Middle Neolithic populations showed a limited genetic impact in this region. A similar genetic shift has been identified by an exhaustive analysis based on haplogroup H [40], showing a minimal genetic continuity between Early Neolithic and Middle/Late Neolithic groups in Central Europe, which the authors consider ‘a previously unrecognised major genetic transition’ [40].

Several scenarios have been proposed to account for this genetic shift between Early/Middle and Late Neolithic in Central Europe, suggesting an influence of the CWC (Corded Ware culture) from the East and of the BBC (Bell Beaker culture) from the West in the Late Neolithic. The impact of people of the CWC culture, in turn massively influenced by a possible influx of populations from the East from the Yamnaya culture, has been proposed to be especially important [41]. While this idea is certainly possible, none of the models studied to date have taken into consideration another possible and obvious explanation, namely a new wave of Neolithic migration into Europe through the ‘traditional route’ of the Balkan Peninsula. This new wave of Neolithic migrations are represented by Vinča and Dudeşti cultures (5500–5000 BC), that trace their origin in North-West Anatolia on the basis of ceramics features [28]. The Boian, Zau and Gumelniţa cultures from Middle-Late Neolithic (M_NEO) from Romania are the direct continuation of this cultural complex; the M_NEO group from Romania displayed differences in haplotype (S5 Fig) and haplogroup distributions (S4 Fig) with the Middle Neolithic from Central Europe.

Interestingly, the genetic analysis of a relatively large number of samples of Boian, Zau and Gumelniţa cultures in Romania (n = 41) (M_NEO) identified a close genetic proximity between this Neolithic group and the Eastern and Central extant European populations. This was shown in the multivariate analysis, where M_NEO and modern populations from Romania are very close, in contrast with Middle Neolithic and modern populations from Central Europe (Figs 2 and 3). Whereas the genetic analysis of modern populations from Central Europe showed a limited genetic impact of the E_NEO_CE and M_NEO_CE groups in this region [21], the mtDNA data of the M_NEO groups from Romania suggest a high genetic impact on modern population in this region (see S2 Fig for shared polymorphisms). The above mentioned data allow us to suggest that the populations of this putative second wave of Neolithic migration from Anatolia caused a much stronger impact on the genetic make-up of the European populations than the earlier farmers of the Starčevo-Criş and LBK cultures.

This hypothesis is supported by the larger number of archaeological sites for the Middle/Late Neolithic and Eneolithic cultures compared with Early Neolithic cultures in South-East Europe, which indicates higher population numbers [28–29]. It is reasonable to hypothesize an interaction of the Vinča-Dudești and Zau-Boian-Gumelniţa cultures with the Late Neolithic cultures of Central Europe. This would have led to gene flow and permeation in Central Europe cultures of mtDNA lineages from the second great Neolithic migrations of South-East Europe, and may have had an important contribution to the genetic shift between Early and Late Neolithic populations in Europe. The hypothesized contribution of Middle Neolithic migrations from North-West Anatolia into the Balkan Peninsula and Central Europe may explain the position of the BBC (Late Neolithic in Central Europe), close to the M_NEO groups from Romania in the multivariate analysis (Figs 2 and 3).

One last aspect concerns the presence of U haplogroups in four individuals from two of the Middle/Late Neolithic sites: Curatesti and Sultana-Valea Orbului. While it could be argued that these individuals share a genetic background with European hunter-gatherers [42] that interacted with and adopted farmer lifestyles, more genetic studies to include local hunter-gatherer populations and nuclear DNA are needed to discern such a possibility. On the other hand, it should be pointed out that no statistical differences of mtDNA between the Curatesti and Sultana-Valea Orbului sites and the other Middle/Late Neolithic populations from Romania were detected.

Two Eneolithic (Eneol) individuals from Romania have been analyzed, showing the same mitochondrial haplotype (haplogroup K) (Table 2). These haplotypes are unique, not found in any mtDNA database of ancient populations. The network performed with the haplotypes corresponding to haplogroup K (S6 Fig) showed that the two individuals from the Decea Mureşului site shared polymorphisms with the ancient and present-day populations from the Near East. Although the two individuals from Decea Mureşului are associated to the Suvorovo culture from the North-Pontic steppes [29–32], and this has been suggested to represent the first contact between Transylvania and North-Pontic steppes, we have not found genetic evidence in the present study to support this hypothesis.

### Bronze Age and the influence of migrations from the East

The archeological data from the Bronze Age in the central Transylvanian plateau of Romania describe at least three major cultures, two of them probably originating and being related to cultures from the East: 1) the Early Bronze Age represented by Copăceni group in the Floreşti-Polus site, which is related to the Yamnaya culture [29]; and 2) the Late Bronze Age complex from Floreşti-Polus site which is related to the Noua-Sabatinovka culture from the North of Black Sea [29]. The most representative number of samples (n = 9) corresponded to the Late Bronze Age (L_BA_Romania). This sample showed a closer genetic similarity with the Bronze Age population from Ukraine than to any other ancient population from Romania. Both F_ST_ distance (S3 Table) and multidimensional scaling analysis (Figs 2 and 3) showed significant differences between Late Bronze Age and Middle Neolithic from Romania, although both populations shared two haplotypes corresponding to haplogroup U5 (ht12 and 13) (Table 2). These results could reflect the influence of migrations from the East into the Bronze Age population of Romania. On the other hand, the unusual mtDNA haplogroup distribution [(H (37.5%), U (25%), HV (25%), W (12.5%)], described in the L_BA_Romania group and the genetic distance to the modern Romania population (Fig 3), suggest that the contribution of L_BA_Romania to the present-day Romanians was relatively limited. Nevertheless, studies on more individuals are necessary to draw definitive conclusions. Also, the impact of the early Bronze Age migrations on the modern South-East Europeans cannot be assessed in our study, due to the low number of samples.

Finally, in this study we report genetic information on the Neolithic and Bronze Age populations of the Balkan Peninsula, a crucial piece of the puzzle integrating the major demographic and cultural changes that took place from the Neolitic period onwards in South-East Europe. Based on aDNA studies from sites of the Starčevo-Criş culture (Cârcea/Gura Baciului/Negrileşti sites), we confirm their genetic relationship with the LBK culture, both originating in the Proto-Sesklo cultures of Northern Greece. In addition, our data support the strong genetic differences between these first European farmers and the later Neolithic farmers. In addition, we provide for the first time a glimpse to the genetic make-up of the farmers from a later Neolithic migration from Anatolia Vinča and Dudești cultures that later evolved in the Boian, Zau and Gumelniţa cultures in South-East Europe. The strong genetic resemblance of individuals from these cultures with the modern populations leads us to propose the hypothesis that they had an important contribution to the genetic heritage of Eastern and Central Europeans. In contrast, no such influence could be demonstrated for Late Bronze Age migrations.

All in all, these data leads to the hypothesis that the Early to Middle/Late Neolithic genetic transition in South-East Europe was strongly influenced by a second migration of farmers from Anatolia during the Middle Neolithic. This scenario may thus lead to a model in which a cultural diffusion process initially brought into Central Europe by small numbers of farmers of the Starčevo-Criş and LBK cultures was later accompanied by a demic expansion of larger numbers of immigrant farmers of the Vinča-Dudeşti and Boian-Gumelniţa cultures. Additional studies are needed in order to define in detail the Neolithic processes of migration in South-East Europe, including an assessment of the local Mesolithic populations, and a more extensive study assessment of Neolithic and Bronze Age Balkan cultures.

## Materials and Methods

### Populations

A mtDNA analysis of a total of 63 individuals recovered from ten sites located in Northern and Southern Romania was carried out; the chronology of these sites ranges from Early Neolithic to the Late Bronze Age. The Early Neolithic (6500–5500 cal BC) sites of Cârcea (Dolj county), Negrileşti (Galaţi county) and Gura Baciului (Cluj county) are associated to the Starčevo-Criş culture (VI millennium BC). Another five sites correspond to Middle/Late Neolithic and Eneolithic period (5500–3800 cal BC): Iclod (Cluj county), Vărăşti (Călăraşi county), Curăteşti (Călăraşi county), Sultana-Malu Roşu (Călăraşi county) and Sultana-Valea Orbului (Călăraşi county). The Late Eneolithic period (4500–3800 BC) is represented by Decea Mureşului site (Alba county), and finally the Bronze Age period by the site of Floresti-Polus (Cluj county). These ten sites were put into several cultural and chronological groups, in order to characterize changes in the mtDNA variability from Early Neolithic (E_NEO) to Late Bronze Age (L_BA) (Fig 1 and Table 1, S7 Fig).

### Early Neolithic (E_NEO_ROMANIA) (6500–5500 cal BC)

Five individuals from the Early Neolithic Romania period come from three sites: 1) Cârcea site is located on the banks of the Cârcea River. Most of the human bones were found in the settlement's "defense ditch" and they were among ceramic fragments and animal bones [43]. 2) Negrileşti is a grave found at 2.90 m depth. The skeleton lying on the right side with bent legs carried on the abdomen and the chest a deposit of snails and a stone [44]. 3) Gura Baciului burials consisted of an inhumation and incineration pit where seven skeletons were inhumated in a bent position [45–46].

### Middle/Late Neolithic and Eneolithic (M_NEO_ROMANIA) (5500–3800 cal BC)

Forty-five samples from individual graves have been recovered from five Middle and Late Neolithic sites. From geographical point of view most of these sites are placed in southeaster area of Romania, near Danube River (Vărăşti) or on the high terrace of Mostiştea Lake (Curăteşti, Sultana-Malu Roşu, Sultana-Valea Orbului). The only exception is the Iclod cemetery that is located in Transylvania, on the banks of the Someşul Mic River. In terms of cultural framework, Iclod cemetery belongs to Zau culture [47–48]; Curăteşti and Sultana-Valea Orbului to Boian culture [49], and Vărăşti and Sultana-Malu Roşu are settlements belonging to Boian and Gumelniţa communities using the same cemetery[49].

### Eneolithic (Eneol_ROMANIA) (4500–3800 cal BC)

This period is represented by Decea Mureşului site (Alba county), dated in the end of the 5th millennium BC. Samples for mtDNA analysis were taken from two of the discovered graves. Exceptional grave goods and the use of ocher and stone mace-head, represent the first contact (migration) between Transylvania and North-Pontic steppes [50].

### Early Bronze Age (E_BA ROMANIA) (2600–2100 cal BC)

Two samples were taken from the great barrow/tumulus from Floresti-Polus (Cluj county) [51–52]. This funerary complex belongs to Copaceni group, dating from the period II of the Early Bronze Age in Transylvania. The Yamnaya culture (Pit-Grave culture) [53–54], that influence this group, appears at the end of 4th millennium BC in the north steppes of the Black Sea [55] and, later it cover a large area to the west, including Transylvania.

### Late Bronze Age (L_BA ROMANIA) (1500–1050 cal BC)

Nine samples for mtDNA analysis come from eight graves from Floresti-Polus (the largest necropolis of Noua culture from Transylvania) [51]. The local populations contributed to cultural genesis of this archaeological complex (Monteoru and Komarov cultures from Moldavia and some eastern contribution—most often attributed to the Iranian people (ancestors cimirienilor, scythians) who, in the second millennium BC dominated a Ponto-Caspian steppes) [56].

### DNA isolation and genetic studies

The processing of the ancient samples in the laboratory involved the application of a series of strict criteria for the authentication of results, detailed in [57–60]. In our case, the extraction and preparation of the PCR was undertaken in a specific lab for aDNA, which consist in a positive-pressure sterile chamber, located in a physically separated space from the laboratory where post-PCR processes are carried out. All the work surfaces were cleaned regularly with sodium hypochlorite and irradiated with UV light. Suitable disposable clothing was worn (lab coat, mask, gloves and cap). Contamination controls were applied in both the extraction and amplification processes.

Selection of samples for performing the present study was made from teeth without caries or deep fissures that might extend into the pulp. Whenever possible, more than one tooth was taken from each individual for duplicate analysis, with the duplicates being analysed in various sessions by different researchers at the University of the Basque Country (UPV/EHU).

In order to eliminate surface contamination, the teeth were subjected to a process of depurination using acids, and the entire surface was irradiated with ultraviolet light [61]. The extraction process followed the protocol described by [62]: the tissue (root of the tooth or powdered bone) was incubated with stirring for 2 hours at 56°C in a lysis buffer (5 ml) (0.5 M EDTA pH 8.0–8.5; 0.5% SDS; 50 mM Tris HCl pH 8.0; 0.01 mg/ml proteinase K). The DNA was recovered using phenol and chloroform and then concentrated and purified (Centricon-30, Amicon). Each extraction session involved two contamination controls that were applied to the entire process, except no dental or bone tissue was added.

### Analysis of mtDNA variability

Sequencing of HVR-I [nucleotide positions (nps) 15,998–16,400] and HVR-II (nps 16504–429) as per [63], was undertaken in six overlapping fragments, each with a length of approximately 100 bp (base pair). Besides, the fragment between primers 8F and 8R [12] was amplified in all samples to determine position 73 of HVR-II of the mtDNA. The amplification of each fragment was undertaken in independent PCRs. In the case of positive amplification and the absence of contamination, the amplifications were purified by ExoSAP-IT (USB Corporation), with subsequent sequencing in an ABI310 automatic sequencer using chemistry based on BigDye 1.1 (Life Technology).

The results obtained were edited with BioEdit software (http://www.mbio.ncsu.edu/BioEdit/bioedit.html) and the sequences were aligned manually. The sequences obtained in the present study are deposited in Genbank under accession numbers KR149064-KR149120.

In order to classify the mitochondrial variability of the individuals analyzed in this study, we proceeded to amplify 11 markers, which are required for defining the 10 Caucasian haplogroups described [64]. The protocol and primers are described in [65]. The digestion patterns were verified using a fragment analyzer (Bioanalyzer, Agilent Technologies).

### Authentication methods

In addition to the precautions taken to avoid contamination, other authentication criteria such as duplication, quantification, cloning and sequencing were applied.

Duplication: A duplicate analysis was performed for 10.3% of individuals at different times and by different researchers at the University of the Basque Country (UPV/EHU).

Quantification of target DNA: Amplifiable DNA was quantified by means of the quantitative PCR (qPCR) of a fragment of 113 bp length of HVR-I, using Taqman probe [66].

Cloning: In order to detect possible heterogeneities in the PCR products that may correspond to either post-mortem damage and/or mixed contamination, a fragment of HVR-I was cloned by means of the TOPO TA Cloning Kit (Invitrogen). Linkage to the vector pCR2.1-TOPO and chemical transformation of the cells TOP10F’ (One Shot E. coli) were performed following the supplier’s instructions [12] (S6 Table).

We have determined the HVR-I and HVR-II sequence of the mtDNA of the researchers and archaeologists who handled the samples in order to discard possible contamination (S5 Table).

Confirmation of the haplogroups obtained by sequencing and cloning of the HVR I of the mtDNA was verified by identifying the SNPs of the coding region by PCR-RFLPs.

In order to identify any possible contamination that might occur in the various stages of the genetic analysis, at least two blanks were included in each extraction round with a control of the PCR in each amplification reaction. If any contamination was detected, the results obtained were discarded.

### Statistical Analysis

Genetic diversity [67] and genetic distances (Fst analysis) were calculated using the statistical package Arlequin 3.11 [68].

Principal Component Analyses (PCAs) was conducted using as variables the frequencies of the mitochondrial haplogroups obtained in this study together with the data from present-day and prehistoric populations taken in the literature (S4 Table) (SPSS 17 Software). In addition, a distance matrix was calculated between the populations studied and those existing in the literature by means of the Arlequin 3.11 program [68]. This distance matrix has been depicted in two dimensions by means of a Multidimensional Scaling (MDS) analysis (SPSS 17 Software).

Furthermore, a Median-Joining Network (MJN) has been constructed using the sequences of ancient and present-day groups from Romania and some ancient groups from Europe that have so far been published, using the Network 4.6.0.0 program (http://www.fluxus-engineering.com). Given the high mutation rate of HVR-I from mtDNA, we applied the substitution rates obtained by Meyer et al. [69–70] to establish varying mutational weights ranging from 0 to10, for this reason some mutation remove in the networks and the reticulations are reduced.

This manuscript involves field studies of anthropological specimens. All necessary permits were obtained for the described study, which complied with all relevant regulations. The National History Museum of Transylvania and “Francisc I. Rainer" Institute of Anthropology, Romanian Academy, gave us the permission to use these samples. We have not conducted field work on site.

## Supporting Information

S1 Fig Median Joining Network of haplotypes distribution. Data encompass mtDNA HVR-I (position 16024 to 16399). Haplotype distribution of the five Rumanian prehistoric groups (present study): Early Neolithic group (green), Middle/Late Neolithic and Eneolithic group (pink), Eneolithic group (blue), Early Bronze Age (black), Late Bronze Age (yelow).
(BMP)

S2 Fig Median Joining Network of haplotypes distribution. Data encompass mtDNA HVR-I (position 16024 to 16399). Haplotype distribution of the Middle/Late Neolithic and Eneolithic group from Romania (present study, S2 Table) and present-day Romania population (28). Middle-Late Neolithic group (pink) and present-day Romania (yellow).
(BMP)

S3 Fig Comparison of the mtDNA haplogroup frequency between LBK (*Linearbandkeramik*, Early Neolithic culture from Central Europe) [21] and Carcea/Gura/precris (Early Neolihic culture in Romania) (present study) populations. (TIF)

S4 Fig Comparison of the mtDNA haplogroup frequency between groups of Middle-Late Neolithic from Central Europe [21] and that of Middle/Late Neolithic and Eneolithic group from Romania (present study). (BMP)

S5 Fig Median Joining Network of haplotypes distribution of the Middle-Late Neolithic groups from Romania (yellow) (present study) and those from Central Europe (blue) [21]. Data encompass mtDNA HVR-I (nps 15999–16399).
(TIF)

S6 Fig Median Joining Network of haplotypes distribution of haplogroup K. Data encompasses mtDNA HVR-I (position 16024 to 16399). Haplotype distribution of the three Romanian prehistoric groups (present study): Middle/Late Neolithic and Eneolithic group (pink), Eneolithic group (light blue), Early Bronze Age (dark blue). Farmers from Near Eastern (lilac) [22] and from Czech Republic (orange) [39]. Present-day populations from: Romania (black), East of Europe (yellow), Near Eastern (green) (S4 Table).
(BMP)

S7 Fig Comparison of the mtDNA haplogroup frequency between: Late Bronze Age group from Romania (present study) and Bronze Age group from Ukraine [37]. (TIF)

S8 Fig Radiocarbon dating of human bone samples analysed in the present study. (TIF)

S1 Table A general chronology of sites analysed in the present study. (DOCX)

S2 Table mtDNA results from 63 ancient individuals from Romania, haplotype of HVR-I and HVR-II, SNPs of the coding region, number of molecules per microlitre and haplotype and haplogroup assignation. HVR-I: Hypervariable Region I of mtDNA (the sequence range from position 16024 to 16399) and HVR-I: Hypervariable Region II of mtDNA (the sequence range from position 0 to 340). rCRS: revised Cambridge Reference Sequence. The figures correspond to the position in region I and II of HVR of mtDNA that changes with respect to the rCRS.
(DOCX)

S3 Table The F_ST_ analysis. a) p-values with standard deviation (p±de) based on the haplotypes frequencies (below the diagonal) and p-values with standard deviation (p±de) based on haplogroup frequencies (upper the diagonal) (P<0.0027, in grey). b) F_ST_ values based on the haplotypes frequencies (below the diagonal) and F_ST_ values based on haplogroup frequencies (upper the diagonal) Ancient samples from Romania: Early Neolithic (E_NEO), Middle Neolithic (M_NEO), Late Neolithic (L_NEO), Early Bronze Age (E_BA), Late Bronze Age (L_BA); Present-day Romanian population (ROM) (Hervella et al., 2014).
(DOCX)

S4 Table Prehistoric and present-day populations compiled from literature constituting the database of HVR-I sequences of mtDNA for the present study. (DOCX)

S5 Table Mitochondrial haplotypes (HVR-I and HVR-II) of researchers and archaeologists. (DOCX)

S6 Table Mitochondrial DNA sequences of the clones from the samples analyzed in the present study. (XLSX)
