# Supplementary figures and images for: Ancient DNA from South-East Europe Reveals Different Events during Early and Middle Neolithic Influencing the European Genetic Heritage
Source: PLoS One. 2015 Jun 8;10(6):e0128810. doi: 10.1371/journal.pone.0128810 (PMC4460020; doi:10.1371/journal.pone.0128810)

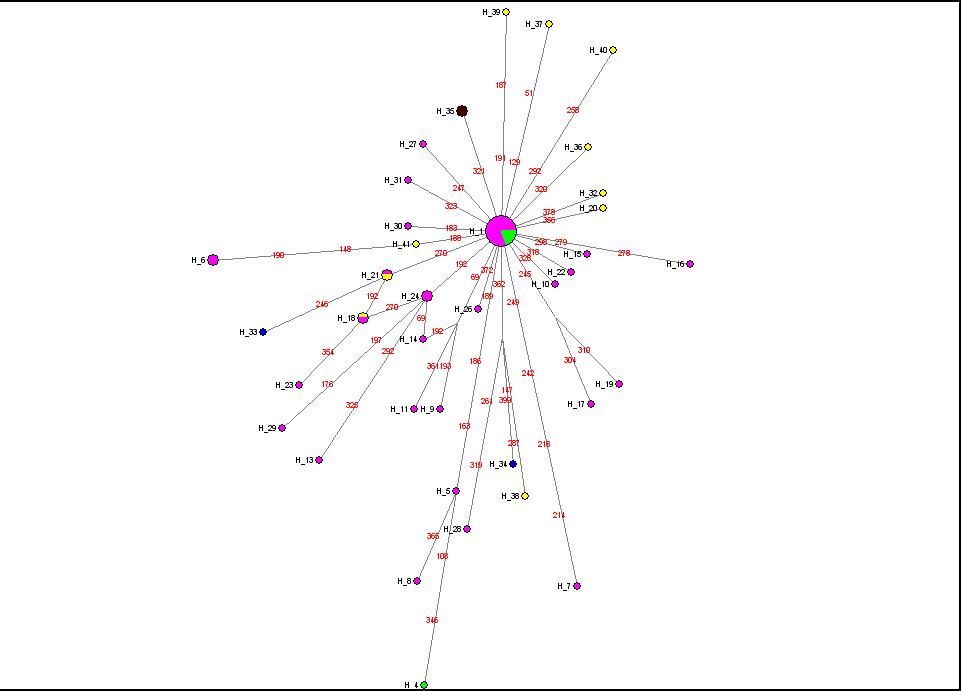

Supplement: S1 Fig — Data encompass mtDNA HVR-I (position 16024 to 16399). Haplotype distribution of the five Rumanian prehistoric groups (present study): Early Neolithic group (green), Middle/Late Neolithic and Eneolithic group (pink), Eneolithic group (blue), Early Bronze Age (black), Late Bronze Age (yelow). (BMP) [file pone.0128810.s001.bmp]

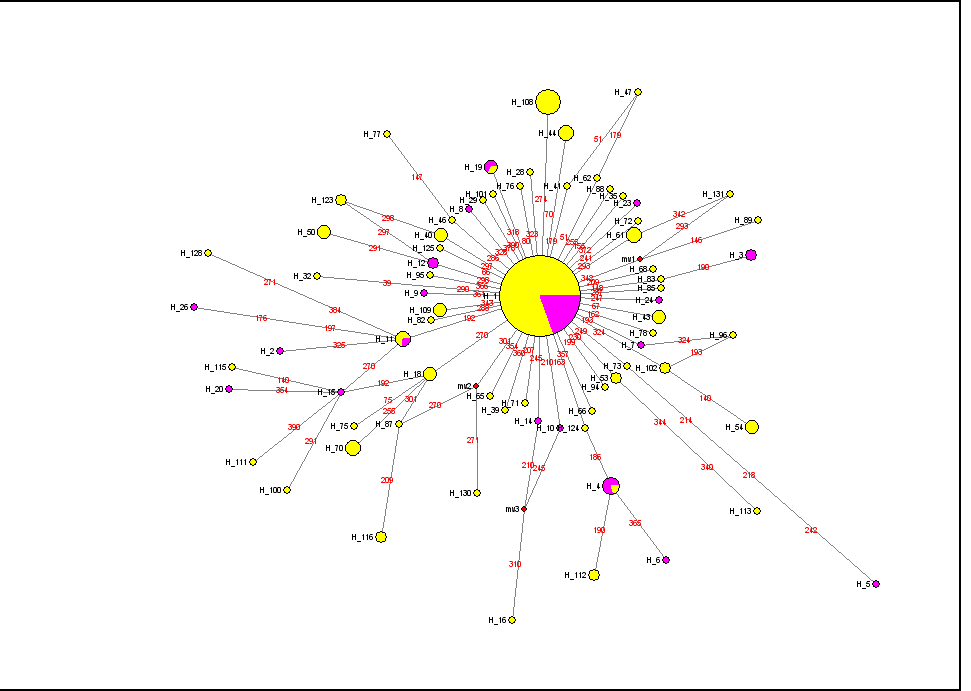

Supplement: S2 Fig — Data encompass mtDNA HVR-I (position 16024 to 16399). Haplotype distribution of the Middle/Late Neolithic and Eneolithic group from Romania (present study, S2 Table) and present-day Romania population (28). Middle-Late Neolithic group (pink) and present-day Romania (yellow). (BMP) [file pone.0128810.s002.bmp]

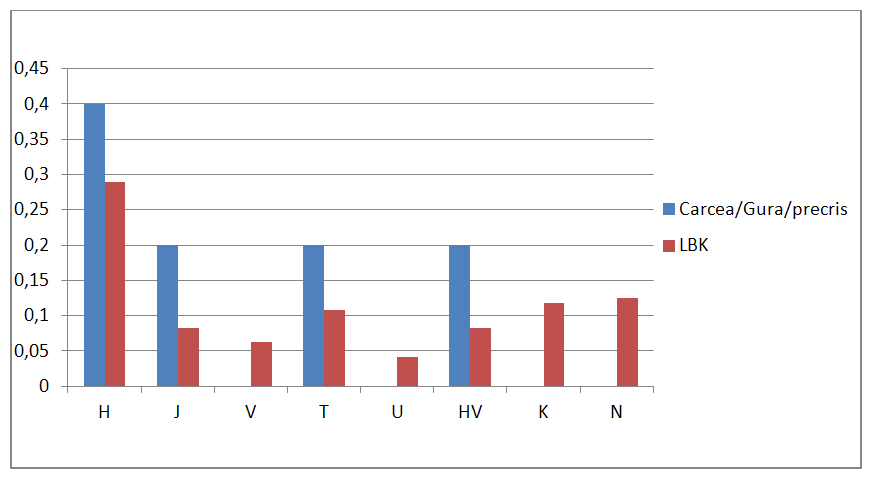

Supplement: S3 Fig — (TIF) [file pone.0128810.s003.tif]

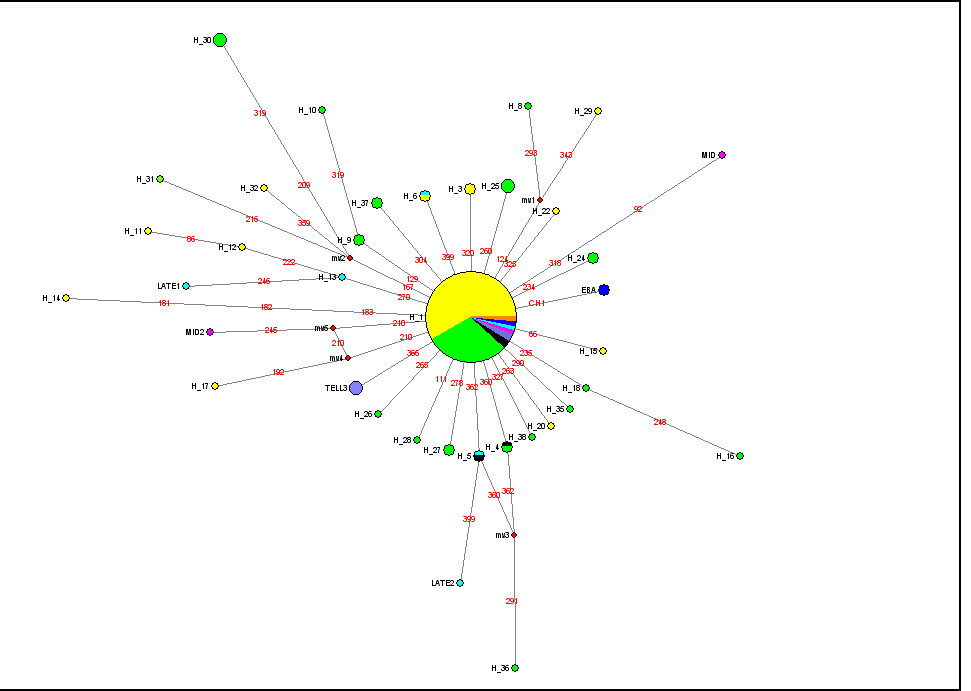

Supplement: S4 Fig — (BMP) [file pone.0128810.s004.bmp]

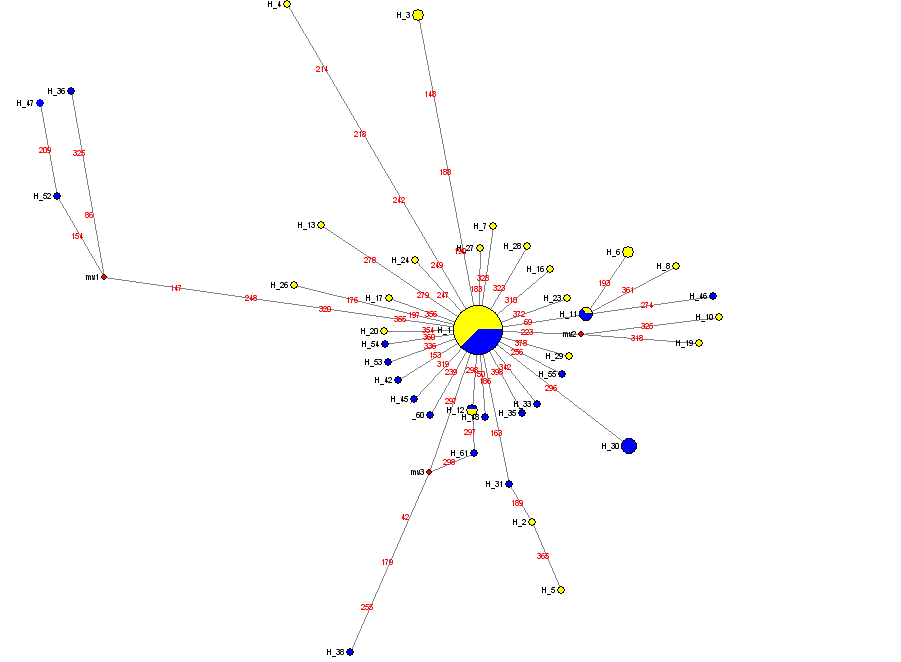

Supplement: S5 Fig — Data encompass mtDNA HVR-I (nps 15999–16399). (TIF) [file pone.0128810.s005.tif]

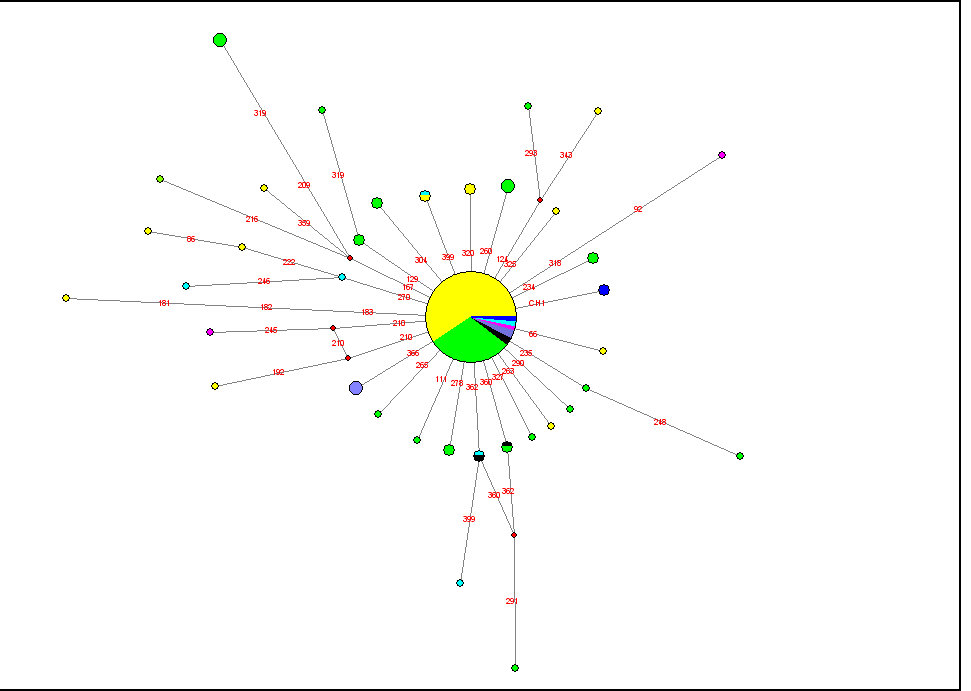

Supplement: S6 Fig — Data encompasses mtDNA HVR-I (position 16024 to 16399). Haplotype distribution of the three Romanian prehistoric groups (present study): Middle/Late Neolithic and Eneolithic group (pink), Eneolithic group (light blue), Early Bronze Age (dark blue). Farmers from Near Eastern (lilac) [22] and from Czech Republic (orange) [39]. Present-day populations from: Romania (black), East of Europe (yellow), Near Eastern (green) (S4 Table). (BMP) [file pone.0128810.s006.bmp]

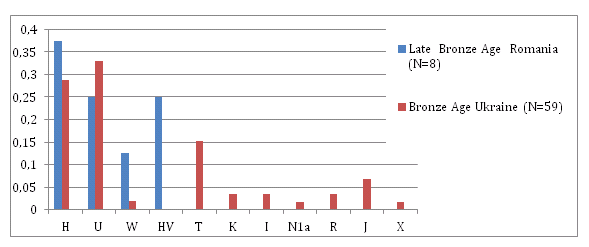

Supplement: S7 Fig — (TIF) [file pone.0128810.s007.tif]

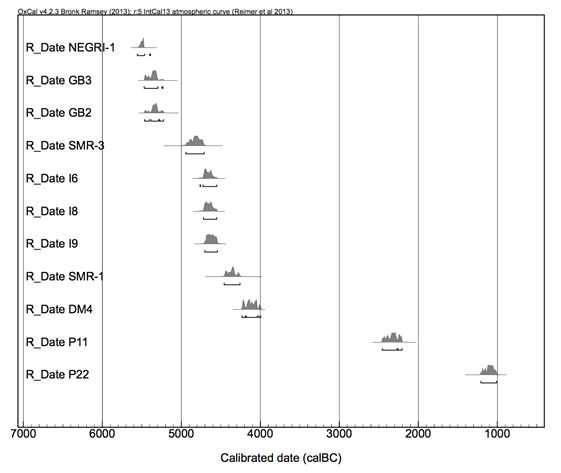

Supplement: S8 Fig — (TIF) [file pone.0128810.s008.tif]
