# Supplementary material for: Ancient DNA from South-East Europe Reveals Different Events during Early and Middle Neolithic Influencing the European Genetic Heritage
Source: PLoS One. 2015 Jun 8;10(6):e0128810. doi: 10.1371/journal.pone.0128810 (PMC4460020; doi:10.1371/journal.pone.0128810)
Supplement: S1 Table — (DOCX) [file pone.0128810.s009.docx]

**S1 Table.** A general chronology of sites analysed in the present study

| **Site** | **Location** | **Site type** | **Period / Datation** | **Culture/Group** |
| --- | --- | --- | --- | --- |
| Cârcea | Dolj county | Settlement | Early Neolithic/  6500–5500 BC | Gura Baciului-Cârcea (Precriș) |
| Gura Baciului | Cluj county | Settlement |  | Starčevo-Criş |
| Negrilești | Galați county | Settlement |  | Starčevo-Criş |
| Curătești | Călărași county | Cemetery | Middle/Late Neolithic and Eneolithic  5500 - 4500 BC | Boian |
| Sultana-Valea Orbului | Călărași county | Cemetery |  | Boian |
| Vărăști | Călărași county | Cemetery |  | Boian- Gumelnița |
| Sultana-Malu Roșu | Călărași county | Cemetery |  | Boian- Gumelnița |
| Iclod | Cluj county | Cemetery |  | Zau |
| Decea Mureșului | Cluj county | Cemetery | Eneolithic/  4500 - 3800 BC | Decea Mureșului |
| Floreşti - Polus | Cluj county | Cemetery | Early Bronze Age II  2600 - 2100 BC | Copăceni |
| Floreşti - Polus | Cluj county | Cemetery | Late Bronze Age  1500 - 1050 BC | Noua |
