# Supplementary material for: Ancient DNA from South-East Europe Reveals Different Events during Early and Middle Neolithic Influencing the European Genetic Heritage
Source: PLoS One. 2015 Jun 8;10(6):e0128810. doi: 10.1371/journal.pone.0128810 (PMC4460020; doi:10.1371/journal.pone.0128810)
Supplement: S2 Table — HVR-I: Hypervariable Region I of mtDNA (the sequence range from position 16024 to 16399) and HVR-I: Hypervariable Region II of mtDNA (the sequence range from position 0 to 340). rCRS: revised Cambridge Reference Sequence. The figures correspond to the position in region I and II of HVR of mtDNA that changes with respect to the rCRS. (DOCX) [file pone.0128810.s010.docx]

**S2 Table.** mtDNA results from 63 ancient individuals from Romania, haplotype of HVR-I and HVR-II, SNPs of the coding region, number of molecules per microlitre and haplotype and haplogroup assignation. HVR-I: Hypervariable Region I of mtDNA (the sequence range from position 16024 to 16400’) and HVR-II: Hypervariable Region II of mtDNA (the sequence range from position 0 to 340’). rCRS: revised Cambridge Reference Sequence. The figures correspond to the position in region I and II of HVR of mtDNA that changes with respect to the rCRS.

| SAMPLE | nºmolec/ul | HVR-I | 73 | HVR-II | HT | *RFLPs* | HG |
| --- | --- | --- | --- | --- | --- | --- | --- |
| GB2 | 226.306 | 16126C-16210G | G |  | ht1 | *+10394DdeI+4216NlaIII* | J |
| GB3 | 3,464.29 | 16311C | A |  | ht2 | *+7025AluI; -14766MseI;-10394DdeI; +4577NlaIII* | HV |
| NE-1 | 3985 | 16136G | A | 263G-309.1C | ht42 | *-14766MseI; -7025AluI* | H |
| Ca1 | 1,979.5 | rCRS | A |  | ht16 | *-14766MseI; -7025AluI* | H |
| Ca2 | 3,832.7 | 16108T-16163G-16186T-16189C-16311C-16346A | G |  | ht17 | *-10394DdeI;+4216NlaIII* | T1a |
| I6 | 7,144.08 | 16126C-16186T-16189C-16294T | G |  | ht3 | *-10394DdeI;+4216NlaIII* | T1a |
| I8 | 4,381.27 | 16069T-16126C-16147T-16188T-16190T-16224C-16311C | G | 73G-146-263G-309.1C-315.1C | ht4 | *+10394DdeI;+4216NlaIII* | J |
| I9 | 2,108.75 | 16069T-16126C-16147T-16188T-16190T-16224C-16311C | G | 73G-146-263G-309.1-315.1C-315.2C | ht4 | *+10394DdeI;+4216NlaIII* | J |
| BV1 | 397.019 | rCRS | A |  | ht16 | *-14766MseI; -7025AluI* | H |
| BV2 | 1,111.47 | 16214T-16218T-16242T-16249C | A |  | ht18 | *-14766MseI; -7025AluI* | H |
| Va1 | 3,964.5 | 16086T-16163G-16189C-16311C-16365T | G |  | ht19 | *-10394DdeI;+4216NlaIII* | T1a |
| Va2 | 1,026.5 | 16069T-16126C-16193T | G |  | ht20 | *+10394DdeI;+4216NlaIII* | J |
| Va3 | 1,220.4 | 16294T-16328T | A |  | ht21 | *-14766MseI; -7025AluI* | H |
| Va4 | 412.17 | rCRS | A |  | ht16 | *-14766MseI; -7025AluI* | H |
| Va5 | 649.76 | 16069T-16126C-16361A | G |  | ht22 | *+10394DdeI+4216NlaIII* | J |
| Va6 | 373.19 | 16210G | A |  | ht23 | *-14766MseI; -7025AluI* | H |
| Va7 | 2,745.8 | 16192T-16223C-16292C-16325C | G |  | ht24 | *-8994HaeII* | W6 |
| Va8 | 2,593.8 | rCRS | A |  | ht16 | *-14766MseI; -7025AluI* | H |
| Va9 | 1,030.3 | 16069T-16126C-16192T-16294T | G |  | ht25 | *+10394DdeI+4216NlaIII* | J |
| Va10 | 235.641 | 16298C | A |  | ht26 | *+7025AluI; -14766MseI;-10394DdeI; +4577NlaIII* | HV |
| Va11 | 235.641 | 16093C-16278T-16279T | A |  | ht27 | *-14766MseI; -7025AluI* | H |
| Va12 | 254.438 | 16245T-16294T-16304C | G |  | ht28 | *-14766MseI; -7025AluI* | H5 |
| Cu1 | 1,150.5 | 16192T-16270T | G |  | ht12 | *+7025AluI;+9052HaeII;+12308HinfI* | U5 |
| Cu2 | 466.1 | 16093C-16210G-16224C-16245T-16311C | G |  | ht29 | *+7025AluI;-9052HaeII;+12308HinfI* | K |
| Su1 | 250.3 | 16356C | G |  | ht30 | *+7025AluI;+9052HaeII;+12308HinfI* | U4 |
| Su2 | - | - | - | - | - | *-* | - |
| Su3 | 270.1 | 16270T | G |  | ht13 | *+7025AluI;+9052HaeII;+12308HinfI* | U |
| Su4 | 326.01 | 16092C-16224C-16318G | G |  | ht31 | *+7025AluI;-9052HaeII;+12308HinfI* | K |
| Su5 | - | - | - | - | - | - | - |
| Su6 | - | - | - | - | - | - | - |
| Su7 | 170.084 | rCRS | A |  | ht16 | *-14766MseI; -7025AluI* | H |
| Su8 | 270.084 | 16192T-16270T-16354T | G |  | ht32 | *+7025AluI;+9052HaeII;+12308HinfI* | U5b |
| Su9 | 303.365 | rCRS | A |  | ht16 | *-14766MseI; -7025AluI* | H |
| Su10 | - | - | - | - | - | - | - |
| Su11 | 90.22 | 16192T | A |  | ht33 | *-14766MseI; -7025AluI* | H |
| Su12 | 90.22 | rCRS | A |  | ht16 | *-14766MseI; -7025AluI* | H |
| Su13 | 453.6 | 16093C-16311C | A |  | ht34 | *+7025AluI;+9052HaeII;+12308HinfI* | U |
| Su14 | 987.6 | 16092C-16371G | A |  | ht35 | *-14766MseI; -7025AluI* | H |
| Su15 | 658.8 | 16247G | A |  | ht36 | *-14766MseI; -7025AluI* | H |
| Su16 | 356.6 | rCRS | A |  | ht16 | *-14766MseI; -7025AluI* | H |
| SMR-1 | 1,503.8 | rCRS | A |  | ht16 | *-14766MseI; -7025AluI* | H |
| SMR-2 | 1,632.2 | 16261T-16319A-16362C | A |  | ht37 | *+12704MboII* | R |
| SMR-3 | 865.4 | rCRS | A |  | ht16 | *-14766MseI; -7025AluI* | H |
| SMR-4 | 965.3 | 16176T-16192T-16197T | A |  | ht38 | *-14766MseI; -7025AluI* | H |
| SMR-5 | 789.3 | 16192T | A |  | ht33 | *-14766MseI; -7025AluI* | H |
| SMR-6 | 368.36 | rCRS | A |  | ht16 | *-14766MseI; -7025AluI* | H |
| SMR-7 | 2458.9 | 16183G | A |  | ht39 | *-14766MseI; -7025AluI* | H |
| SMR-8 | 789.6 | rCRS | A |  | ht16 | *-14766MseI; -7025AluI* | H |
| SMR-9 | 986.5 | 16294T-16323C-16311C | A |  | ht40 | *-14766MseI; -7025AluI* | H |
| SMR-10 | 1,596.3 | 16379T | A |  | ht41 | *-14766MseI; -7025AluI* | H |
| DM3 | 289.879 | 16093C-16224C-16246G-16270T-16311C | G |  | ht5 | *+7025AluI;-9052HaeII;+12308HinfI* | K |
| DM4 | 1,918.15 | 16093C-16224C-16311C-16362C-16399G | G |  | ht6 | *+7025AluI;-9052HaeII;+12308HinfI* | K |
| P11 | 24,603.7 | 16224C-16311C-16321T | G | 73G-263G-309.1C-315.1C | ht7 | *+7025AluI;-9052HaeII;+12308HinfI* | K |
| P12A | 868.813 | 16224C-16311C-16321T | G | 73G-263G-309.1C-315.1C-315.2C | ht7 | *+7025AluI;-9052HaeII;+12308HinfI* | K |
| P22 | 42,246.4 | 16320T | G | 73G-263G-315.1C | ht8 | *+8249avaII; -8250HaeII;, -8994HaeIII; -12704MboI; I-4216NlaIII* | W |
| P23 | 20,357.5 | 16320T | G | 73G-263G-315.1C | ht8 | *+8249avaII; -8250HaeII;, -8994HaeIII; -12704MboII; -4216NlaIII* | W |
| P24 | 11,885.6 | 16051G-16129A | A |  | ht9 | *-14766MseI; -7025AluI; +6776Tsp509I* | H1 |
| P25 | 7,207.53 | 16147T-16224C-16287T-16362C | A |  | ht10 | *-14766MseI; -7025AluI* | H |
| P26 | 9,093.35 | 16191T-16187T | A |  | ht11 | *+7025AluI; -14766MseI;-10394DdeI; +4577NlaIII* | HV |
| P27 | 9,279.0 | 16192T-16270T | G |  | ht12 | *+7025AluI;+9052HaeII;+12308HinfI* | U5 |
| P28 | 3,123.43 | 16270T | G |  | ht13 | *+7025AluI;+9052HaeII;+12308HinfI* | U5 |
| P29 | 4,528.05 | 16210G-16292T | A |  | ht14 | *+7025AluI; -14766MseI;-10394DdeI; +4577NlaIII; +12704MboII* | HV |
| P30 | 66,906.7 | 16188T-16311C | A |  | ht15 | *-14766MseI; -7025AluI* | H |
