# Supplementary material for: Ancient DNA from South-East Europe Reveals Different Events during Early and Middle Neolithic Influencing the European Genetic Heritage
Source: PLoS One. 2015 Jun 8;10(6):e0128810. doi: 10.1371/journal.pone.0128810 (PMC4460020; doi:10.1371/journal.pone.0128810)
Supplement: S3 Table — a) p-values with standard deviation (p±de) based on the haplotypes frequencies (below the diagonal) and p-values with standard deviation (p±de) based on haplogroup frequencies (upper the diagonal) (P<0.0027, in grey). b) FST values based on the haplotypes frequencies (below the diagonal) and FST values based on haplogroup frequencies (upper the diagonal) Ancient samples from Romania: Early Neolithic (E_NEO), Middle Neolithic (M_NEO), Late Neolithic (L_NEO), Early Bronze Age (E_BA), Late Bronze Age (L_BA); Present-day Romanian population (ROM) (Hervella et al., 2014). (DOCX) [file pone.0128810.s011.docx]

**S3 Supplementary Table.** The F_ST_ analysis: a) *p*-values with standard deviation (p±de) based on the haplotypes frequencies (below the diagonal) and *p*-values with standard deviation (p±de) based on haplogroup frequencies (upper the diagonal) (P<0.0027, in grey). b) F_ST_ values based on the haplotypes frequencies (below the diagonal) and F_ST_ values based on haplogroup frequencies (upper the diagonal) Ancient samples from Romania: Early Neolithic (E_NEO), Middle Neolithic (M_NEO), Late Neolithic (L_NEO), Early Bronze Age (E_BA), Late Bronze Age (L_BA); Present-day Romanian population (ROM) (Hervella et al., 2014).

**a)**

|  | **E_NE** | **M_NE** | **L_NE** | **E_BA** | **L_BA** | **ROM** |
| --- | --- | --- | --- | --- | --- | --- |
| **E_NE** | ***** | 0.00000±0.0000 | 0.00000±0.0000 | 0.00000±0.0000 | 0.00000±0.0000 | 0.00000±0.0000 |
| **M_NE** | 0.000901±0.0091 | ***** | 0.00000±0.0000 | 0.00000±0.0000 | 0.00000±0.0000 | 0.810810±0.0489 |
| **L_NE** | 0.00000±0.0000 | 0.00000±0.0000 | ***** | 0.01560±0.06589 | 0.00000±0.0000 | 0.00000±0.0000 |
| **E_BA** | 0.00000±0.0000 | 0.00000±0.0000 | 0.09009±0.03030 | ***** | 0.00000±0.0000 | 0.00000±0.0000 |
| **L_BA** | 0.00000±0.0000 | 0.00000±0.0000 | 0.00000±0.0000 | 0.00000±0.0000 | ***** | 0.00000±0.0000 |
| **ROM** | 0.00000±0.0000 | 0.00000±0.0000 | 0.00000±0.0000 | 0.00000±0.0000 | 0.00000±0.0000 | ***** |

b)

|  | **E_NE** | **M_NE** | **L_NE** | **E_BA** | **L_BA** | **ROM** |
| --- | --- | --- | --- | --- | --- | --- |
| **E_NE** | ***** | -0.03184 | 0.20705 | 0.20705 | -0.07256 | -0.03584 |
| **M_NE** | 0.07673 | ***** | 0.41805 | 0.41805 | 0.01768 | -0.00978 |
| **L_NE** | 0.24579 | 0.16725 | * | 0 | 0.36661 | 0.38161 |
| **E_BA** | 0.43860 | 0.32295 | 0.66667 | * | 0.36661 | 0.38161 |
| **L_BA** | 0.14967 | 0.09488 | 0.18220 | 0.34646 | * | -0.00448 |
| **ROM** | 0.07968 | 0.03360 | 0.12318 | 0.28353 | 0.06219 | ***** |
