# Supplementary material for: Ancient DNA from South-East Europe Reveals Different Events during Early and Middle Neolithic Influencing the European Genetic Heritage
Source: PLoS One. 2015 Jun 8;10(6):e0128810. doi: 10.1371/journal.pone.0128810 (PMC4460020; doi:10.1371/journal.pone.0128810)
Supplement: S4 Table — (DOCX) [file pone.0128810.s012.docx]

**S4 Table.** Present-day and ancient populations compiled from literature constituting the database of HVR-I sequences of mtDNA for the present study.

| Abbrev**.** | **Populations** | **References** |
| --- | --- | --- |
| MdE | Greece | (1-2) |
|  | Albania |  |
| MdC | Italy | (1,3-4) |
|  | Rome |  |
|  | Sicily |  |
|  | Sardinia |  |
|  | Gipuzkoa | (1, 5-10) |
|  | Bizkaia |  |
| Cantabrian Fringe | Álava |  |
|  | Basque Country |  |
|  | Galicia |  |
|  | Asturias |  |
|  | Cantabria |  |
| Alps | Switzerland | (1,11) |
|  | Germany South |  |
|  | Poland | (1,1-12) |
|  | Czechoslovakia |  |
|  | Germany |  |
| NC | Denmark |  |
|  | Switzerland | (1, 13) |
| SCA | Norway |  |
| NW | France | (1, 14-15) |
|  | England |  |
|  | Scotland |  |
| NE | Estonia, Finnland | (1, 28) |
| IP | Andalucía | (6,16-19) |
|  | Castilla-León |  |
|  | Portugal |  |
|  | Catalonia |  |
| Bulgaria |  | (20) |
| Romania |  | (29) |
| Hungary |  | (24) |
| Bosnia |  | (23) |
| Czech Republic |  | (22) |
| Russia |  | (21) |
| N Caucasus | Northern Caucasus | (1,10) |
| Iraq |  | (1) |
| Syria |  | (1) |
| Palestine |  | (1,4) |
| Turkey |  | (1,25-27) |
| Kurdistan |  | (1) |
| Armenia |  | (1) |

| **Abbrev.** | **Location** | **Reference** |
| --- | --- | --- |
| HG_SCA | Scandinavia | [30-32] |
| HG_CE | Central and Eastern Europe | [33,34] |
| HG_Cant_Fringe | Cantabrian Fringe of Spain | [35,36] |
| E_NEO_ Cant_Fringe | Cantabrian Fringe of Spain | [35] |
| L_ NEO_ Cant_Fringe | Cantabrian Fringe of Spain | [36] |
| NEO_Hungary | Hungary | [37] |
| NEO_E_Pyrenees | East of Pyrenees | [38] |
| NEO_France | France | [39] |
| NEO_Catalonia | East Spain | [40] |
| E_NEO_CE | Central and Eastern Europe | [30,37] |
| M_NEO_CE | Central and Eastern Europe | [30] |
| CWC_CE | Central and Eastern Europe | [30] |
| BBC_CE | Central and Eastern Europe | [30] |

**References**

1 Richards M, Macaulay V, Hickey E, Vega E, Sykes B, et al. (2000) Tracing European founder lineages in the Near Eastern mtDNA pool. Am J Hum Genet 67: 1251-76.

2. Belledi M, Poloni ES., Casalotti R, Conterio F, Mikerezi I, et al. (2000) Maternal and paternal lineages in Albania and the genetic structure of Indo-European populations. Eur J Hum Genet 8: 480-486.

3. Torroni A, Bandelt HJ, D'Urbano L, Lahermo P, Moral P, et al. (1998) mtDNA analysis reveals a major late Paleolithic population expansion from southwestern to northeastern Europe. Am J Hum Genet 62: 1137-52.

4. Di Rienzo A, Wilson, AC (1991) Branching pattern in the evolutionary tree for human mitochondrial DNA. Proc Natl Acad Sci USA 88: 1597-1601.

5. Bertranpetit J, Sala J, Calafell F, Underhill PA, Moral P, et al. (1995) Human mitochondrial DNA variation and the origin of Basques. Ann Hum Genet 59: 63-81.

6. Corte-Real HB, Macaulay VA, Richards MB, Hariti G, Issad MS, et al. (1996) Genetic diversity in the Iberian Peninsula determined from mitochondrial sequence analysis. Ann Hum Genet 60: 331-350.

7. Alfonso-Sánchez MA, Cardoso S, Martínez-Bouzas C, Peña JA, Herrera, RJ, et al. (2008) Mitochondrial DNA haplogroup diversity in Basques: A reassessment based on HVI and HVII polymorphisms. Am J Hum Biol 20: 154-156.

8. García O, Fregel R, Larruga JM, Fregel R, Larruga JM, et al. (2011) Using mitochondrial DNA to test the hypothesis of a European post-glacial human recolonization from the Franco-Cantabrian refuge. Heredity 106: 37-45.

9. Salas A, Comas D, Lareu MV, Bertranpetit J, Carracedo A (1998) mtDNA analysis of the Galician population: a genetic edge of European variation. Eur J Hum Genet 6: 365-75.

10. Maca-Meyer N, Gonzalez AM, Pestano J, Flores C, Larruga JM, et al. (2003) Mitochondrial DNA transit between West Asia and North Africa inferred from U6 phylogeography BMC Genet 4: 15.

11. Pult I, Sajantila A, Simanainen J, Georgiev O, Schaffner W, et al. (1994) Mitochondrial DNA sequences from Switzerland reveal striking homogeneity of European populations. Biol Chem Hoppe Seyler 375: 837-840.

12. Poetsch M, Wittig, H, Krause D, Lignitz E (2003) Mitochondrial diversity of a northeast German population sample. Forensic Sci Int **137:** 125-132.

13. Opdal SH, Rognum TO, Vege A, Stave AK, Dupuy BM, et al. (1998) Increased number of substitutions in the D-loop of mitochondrial DNA in the sudden infant death syndrome. Acta Paediatr 87: 1039-1044.

14. Helgason A, Sigurethardottir S, Gulcher JR, Ward R, et al. (2000) mtDNA and the origin of the Icelanders: deciphering signals of recent population history. Am J Hum Genet 66: 999-1016.

15. Piercy R, Sullivan KM, Benson N, Gill, P (1993) The application of mitochondrial DNA typing to the study of white Caucasian genetic identification. Int J Legal Med 106: 85-90.

16. González AM, Brehm A, Pérez JA, Maca-Meyer N, Flores C, et al. (2003) Mitochondrial DNA Affinities at the Atlantic Fringe of Europe. Am J Phys Anthropol 120: 391-404.

17. Alvarez-Iglesias JC, Johnson DL, Lorente JA, Martinez-Espin E, Martinez-Gonzalez LJ, et al. (2007) Characterization of human control region sequences for Spanish individuals in a forensic mtDNA data set. Leg Med (Tokyo) 9: 293-304.

18. Larruga JM, Diez F, Pinto FM, Flores C, Gonzalez AM (2001) Mitochondrial DNA characterisation of European isolates: the Maragatos from Spain. Eur J Hum Genet 9: 708-16.

19. Plaza S, Calafell F, Helal A, Bouzerna N, Lefranc G, et al. (2003) Joining the Pillars of Hercules: mtDNA sequences show multidirectional gene flow in the western Mediterranean. Ann Hum Genet 67: 312-328.

20. Karachanak S, [Carossa V](http://www.ncbi.nlm.nih.gov/pubmed?term=Carossa%20V%5BAuthor%5D&cauthor=true&cauthor_uid=21674295), [Nesheva D](http://www.ncbi.nlm.nih.gov/pubmed?term=Nesheva%20D%5BAuthor%5D&cauthor=true&cauthor_uid=21674295), [Olivieri A](http://www.ncbi.nlm.nih.gov/pubmed?term=Olivieri%20A%5BAuthor%5D&cauthor=true&cauthor_uid=21674295), [Pala M](http://www.ncbi.nlm.nih.gov/pubmed?term=Pala%20M%5BAuthor%5D&cauthor=true&cauthor_uid=21674295), et al. (2012) Bulgarians vs the other European populations: a mitochondrial DNA perspective*.* Int J Legal Med 126: 497–503.

21. Malyarchuck BA, Grzybowski T, Derenko MV, Czarny J, Woźniak M, et al. (2002) Mitochondrial DNA variability in Poles and Russians. Ann Hum Genet 66: 261–283.

22. Malyarchuk BA, Vanecek T, Perkova M A, Derenko MV, Sip M (2006) Mitochondrial DNA variability in the Czech population, with application to the ethnic history of Slavs. Hum Biol 78: 681–696.

23. Malyarchuk BA, [Perkova MA](http://www.ncbi.nlm.nih.gov/pubmed?term=Perkova%20MA%5BAuthor%5D&cauthor=true&cauthor_uid=18205894), [Derenko MV](http://www.ncbi.nlm.nih.gov/pubmed?term=Derenko%20MV%5BAuthor%5D&cauthor=true&cauthor_uid=18205894), [Vanecek T](http://www.ncbi.nlm.nih.gov/pubmed?term=Vanecek%20T%5BAuthor%5D&cauthor=true&cauthor_uid=18205894), [Lazur J](http://www.ncbi.nlm.nih.gov/pubmed?term=Lazur%20J%5BAuthor%5D&cauthor=true&cauthor_uid=18205894), et al. (2008) Mitochondrial DNA variability in Slovaks, with application to the Roma origin. Ann Hum Genet 72**:** 228-40.

24. Sajantila A, Lahermo P, Anttinen T, Lukka M, Sistonen P, et al. (1995) Genes and languages in Europe: an analysis of mitochondrial lineages. Genome Res 5: 42-52

25. Comas D, Calafell F, Mateu E, Perez-Lezaun A, Bertranpetit J (1996) Geographic variation in human mitochondrial DNA control region sequence: the population history of Turkey and its relationship to the European populations. Mol Biol Evol 13: 1067-77.

26. Calafell F, Underhill P, Tolun A, Angelicheva D, Kalaydjieva L (1996) From Asia to Europe: mitochondrial DNA sequence variability in Bulgarians and Turks. Ann Hum Genet 60: 35-49.

27. Francalacci P, Bertranpetit J, Calafell F, Underhill PA (1996) Sequence diversity of the control region of mitochondrial DNA in Tuscany and its implications for the peopling of Europe. Am J Phys Anthropol 100: 443-460.

28. Sajantila A, [Salem AH](http://www.ncbi.nlm.nih.gov/pubmed?term=Salem%20AH%5BAuthor%5D&cauthor=true&cauthor_uid=8876258), [Savolainen P](http://www.ncbi.nlm.nih.gov/pubmed?term=Savolainen%20P%5BAuthor%5D&cauthor=true&cauthor_uid=8876258), [Bauer K](http://www.ncbi.nlm.nih.gov/pubmed?term=Bauer%20K%5BAuthor%5D&cauthor=true&cauthor_uid=8876258), [Gierig C](http://www.ncbi.nlm.nih.gov/pubmed?term=Gierig%20C%5BAuthor%5D&cauthor=true&cauthor_uid=8876258), et al. (1996) Paternal and maternal DNA lineages reveal a bottleneck in the founding of the Finnish population. Proc Natl Acad Sci U S A 93: 12035-12039.

29. Hervella M, Izagirre N, Alonso S, Iona M, Netea M, et al. (2014) The Carpathian range represents a weak genetic barrier in South-East Europe. BMC Genetics 15: 56.

1. Skoglund P, Malmström H, Raghavan M, Stora J, Hall P, et al. (2012) Origins and genetic legacy of Neolithic farmers and hunter-gatherers in Europe. Science 336: 466-9.
2. Skoglund P, Malmström H, Omrak A, Raghavan M, Vadiosera C, et al. (2014) Genomic diversity and admixture differs for Stone-Age Scandinavian Foragers and farmers. Science 344: 747-750.
3. Malmström H, Gilbert MT, Thomas MG, Brandstrom M, Stora J, et al. (2009) Ancient DNA reveals lack of continuity between Neolithic hunter-gatherers and contemporary Scandinavians. Curr Biol 19: 1758-62.
4. Bramanti B, Thomas MG, Haak W, Unterlaender M, Jores, P, et al. (2009) Genetic discontinuity between local hunter-gatherers and Central Europe's first farmers. Science 326: 137-140.
5. Brandt G, [Haak](http://www.sciencemag.org/search?author1=Wolfgang+Haak&sortspec=date&submit=Submit) W, Adler CJ, Roth C, Szécsényi-Nagy A, et al. (2013) Ancient DNA reveals key stages in the formation of central European mitochondrial genetic diversity. Science 342: 257-261
6. Hervella M, Izagirre N, Alonso S, Fregel R, Alonso A, et al*.* (2012) Ancient DNA from hunter-gatherer and farmer groups from Northern Spain supports a random dispersion model for the Neolithic expansion into Europe. Plos On*e* 7: e34417.
7. Sanchez-Quinto F, [Schroeder](javascript:void(0);) H, Ramirez O, Avila-Arcos MC, Pybus M, et al. (2012) Genomic affinities of two 7,000-year-old Iberian hunter-gatherers*.* Curr Biol 22: 1494-1499.
8. Izagirre N, de la Rúa C (1999) An mtDNA analysis in ancient Basque populations: implications for haplogroup V as a marker for a major paleolithic expansion from southwestern Europe. Am J Hum Genet 65: 199-207.
9. Guba Hadadi E, Major A, Furka T, Juhasz E, et al. (2012) HVS-I polymorphism screening of ancient human mitochondrial DNA provides evidence for N9a discontinuity and East Asian haplogroups in the Neolithic Hungary. J Hum Genet 56: 784-789.
10. Gamba C, Fernandez C, Tirado M, Deguilloux MF, Pemonge MH, et al. (2012) Ancient DNA from an Early Neolithic Iberian population supports a pioneer colonization by first farmers. Mol Ecol 21: 45-56
11. Lacan M, Keyser C, Ricaut FX, [Brucato N](http://www.ncbi.nlm.nih.gov/pubmed?term=%22Brucato%20N%22%5BAuthor%5D), [Duranthon F](http://www.ncbi.nlm.nih.gov/pubmed?term=%22Duranthon%20F%22%5BAuthor%5D), et al. (2011) Ancient DNA reveals male diffusion through the Neolithic Mediterranean route. Pro Natl Acad Sci U S A 108: 9788-9791.
12. Sampietro M L, Lao O, Caramelli DL, Pou R, Marti M, et al. (2007) Palaeogenetic evidence supports a dual model of Neolithic spreading into Europe. Proc R Soc B 274: 2161-2167.
13. Haak W, Forster P, Bramanti B, Matsumura S, Brandt G, et al. (2005) Ancient DNA from the first European farmers in 7500-year-old Neolithic sites. Science 310: 1016-1018.
14. Haak W, Balanovsky O, Sanchez JJ, [Koshel S](http://www.ncbi.nlm.nih.gov/pubmed?term=%22Koshel%20S%22%5BAuthor%5D), [Zaporozhchenko V](http://www.ncbi.nlm.nih.gov/pubmed?term=%22Zaporozhchenko%20V%22%5BAuthor%5D), et al. (2010) Ancient DNA from European early neolithic farmers reveals their near eastern affinities. PLoS Biol 8: e1000536.
