# Supplementary material for: Ancient DNA from South-East Europe Reveals Different Events during Early and Middle Neolithic Influencing the European Genetic Heritage
Source: PLoS One. 2015 Jun 8;10(6):e0128810. doi: 10.1371/journal.pone.0128810 (PMC4460020; doi:10.1371/journal.pone.0128810)
Supplement: S5 Table — (DOCX) [file pone.0128810.s013.docx]

**S5 Table.** Mitochondrial haplotypes (HVR-I and HVR-II) of researchers and archaeologists

| **Lab** | **Researcher** | **HVR I haplotype** | **HVR II haplotype** |
| --- | --- | --- | --- |
| Univ. Basque Country (Spain) |  |  |  |
|  | Researcher #1 | 189 | 263G-315.1C |
|  | Researcher #2 | 092-224-311 | 73G-146C-263G-315.1C |
| Archaeologists |  |  |  |
|  | Researcher #1 | 051-179-356-362 | N.A |
|  | Researcher #2 | 069-126-299 | N.A |
|  | Researcher #3 | 051-312 | N.A |
|  | Researcher #4 | 051-162-264 | N.A |
|  | Researcher #5 | 298 | N.A |
|  |  |  |  |

HVR-I: Hypervariable Region I of mtDNA. rCRS: revised Cambridge Reference Sequence. The figures correspond to the position in region I of HVR of mt DNA that changes with respect to the rCRS. Precise mitochondrial coordinates can be obtained by adding 16.000 (N.A.: not analyzed).
